# Supplementary material for: Identification of circulating proteins associated with general cognitive function among middle-aged and older adults
Source: Commun Biol. 2023 Nov 3;6:1117. doi: 10.1038/s42003-023-05454-1 (PMC10624811; doi:10.1038/s42003-023-05454-1)
Supplement: Supplementary file 2 — Supplementary Information [file 42003_2023_5454_MOESM2_ESM.pdf]

The file contains supplementary notes 1 and 2 and supplementary figures 1 to 31.

Supplementary Note 1

Results of the Mendelian Randomization analysis of eGFR on general cognitive function

Of the 443 proxy SNPs of eGFR after clumping by r2 < 0.001, 173 were also associated with blood urea nitrogen (BUN) in consistent effect direction for kidney function with p< 0.05 and retained for MR analysis. All MR methods did not support kidney function as a potential causal factor of general cognitive function (inverse variance weighted multiplicative random effect [IVW MRE] beta=-0.004, p=9.71E-1, **Supplementary Table 1**).

Supplementary Table 1. Results of MR analysis on the potential causal relationship between kideny function and general cognitive function

Method	# of proxy SNPs used	Beta	SE	P-value	P-value for hetero-geneity	Egger intercept	Egger intercept SE	Egger intercept p-value
IVW-MRE	173	0.004	0.104	9.71E-01	2.08E-11	-7.51E-04	7.57E-04	3.23E-01
MR Egger	173	0.216	0.237	3.65E-01	--	--	--	--
Radial iterative	147	0.004	0.104	9.71E-01	--	--	--	--
Weighted median	173	0.003	0.141	9.81E-01	--	--	--	--
Weighted mode	173	0.027	0.203	8.96E-01	--	--	--	--

Abbreviation. IVW-MRE, inverse variance weighted multiplicative random effect

Supplementary Note 2

Descriptions and Acknowledgments of Discovery Cohorts

ARIC

The Atherosclerosis Risk in Communities (ARIC) study is a prospective cohort study from four communities: Washington County, MD, Jackson, MS, Forsyth, NC, and Minneapolis, MN. A total of 15,792 participants aged 45 to 65 were recruited from 1987 to 1989.[8, 9] The data for the current analysis, including cognitive tests and blood samples for protein assay, were collected at Visit 5 (2011 to 2013). The cognitive tests used to construct the general cognitive function phenotype were the Delayed Word Recall Test (DWRT), an assessment of verbal declarative memory; the Digit Symbol Substitution Test (DSST), a test of information processing speed; and the Word Fluency Test (WFT), a test of phonemic verbal fluency.[10, 11, 12]

The ARIC study has been funded in whole or in part with Federal funds from the National Heart, Lung, and Blood Institute, National Institutes of Health, Department of Health and Human Services (contract numbers HHSN268201700001I, HHSN268201700002I, HHSN268201700003I, HHSN268201700004I and HHSN268201700005I), R01HL087641, R01HL059367 and R01HL086694; National Human Genome Research Institute contract U01HG004402; and National Institutes of Health contract HHSN268200625226C. Funding was also supported by 5RC2HL102419, R01NS087541 and R01HL131136. Neurocognitive data were collected by U01 2U01HL096812, 2U01HL096814, 2U01HL096899, 2U01HL096902, 2U01HL096917 from the NIH (NHLBI, NINDS, NIA and NIDCD). Infrastructure was partly supported by Grant Number UL1RR025005, a component of the National Institutes of Health and NIH Roadmap for Medical Research. The authors thank the staff and participants of the ARIC study for their important contributions. Infrastructure was partly supported by Grant Number UL1RR025005, a component of the National Institutes of Health and NIH Roadmap for Medical Research.

CHS

The Cardiovascular Health Study (CHS) is a population-based cohort study of risk factors for coronary heart disease and stroke in adults ≥65 years conducted across four field centers [PMID: 1669507]. In June 1990, four Field Centers (Sacramento, CA; Hagerstown, MD; Winston-Salem, NC; Pittsburgh, PA) completed the recruitment of 5201 primarily European ancestry participants from random samples of the Medicare eligibility lists. Between November 1992 and June 1993, an additional 687 African Americans were recruited using similar methods for a total sample of 5,888.

Blood samples were drawn from all participants at their baseline examination and during follow-up clinic visits. Between enrollment and 1998-99, participants were seen in the clinic annually, and contacted by phone at 6-month intervals to collect information about hospitalizations and potential cardiovascular events. CHS was approved by institutional review committees at each field center and individuals in the present analysis gave informed consent including consent for the study of cardiovascular disease.

Frozen, stored blood samples from the 1992-3 clinic visit were evaluated for proteomics analysis. SomaScan 5K proteomic assays were performed by SomaLogic.

Cognitive exams were performed at each annual in person study visit. Scores for this analysis were from exam year 6 in order to have three concurrently-administered cognitive exams. CHS cognitive exams used in this analysis are the Benton Visual Retention Test, the Digit Symbol Substitution Test, and the Modified Mini-Mental State Exam.

This Cardiovascular Heath Study (CHS) research was supported by NHLBI contracts HHSN268201200036C, HHSN268200800007C, HHSN268201800001C, N01HC55222, N01HC85079, N01HC85080, N01HC85081, N01HC85082, N01HC85083, N01HC85086, 75N92021D00006; and NHLBI grants U01HL080295, R01HL087652, R01HL105756, R01HL103612, R01HL120393, R01HL085251, R01HL144483, and U01HL130114 with additional contribution from the National Institute of Neurological Disorders and Stroke (NINDS). Additional support was provided through R01AG023629, R01AG15928, and R01AG20098 from the National Institute on Aging (NIA). AEF is supported by K01AG071689. A full list of principal CHS investigators and institutions can be found at CHS-NHLBI.org.
The content is solely the responsibility of the authors and does not necessarily represent the official views of the National Institutes of Health.

FHS Gen3

The design of the Framingham Heart Study (FHS) has been detailed previously.[13, 14, 15] In brief, the study was initiated in 1948 with an enrollment of 5209 mostly Caucasian individuals from the town of Framingham, MA, USA. This cohort is referred to as the Original cohort. Starting in 1971, the offspring of the Original Cohort along with their spouses (n=5124), were enrolled in the 'Offspring cohort' and have been examined at 4-yearly intervals thereafter. In 2002, the children of the Offspring cohort were enrolled as the 'Third generation cohort' or Gen 3 and are examined periodically with a target interval of 4 years between examinations, although for logistic reasons some exams were 6-8 years apart (n=4095). SomaScan proteomic profiling were performed on 1,913 participants from the Offspring cohort using plasma samples collected during the fifth examination cycle (1991-1995) and 900 participants from the Generation 3 (Gen 3) cohort using plasma samples collected during the second examination cycle (2008-2011). Cognitive measures assessed between 1999-2018 for Offspring and 2009-2018 for Gen3 were used in this study. The Framingham Heart Study is conducted and supported by the National Heart, Lung, and Blood Institute (NHLBI) in collaboration with Boston University (Contract No. N01-HC-25195, HHSN268201500001I and 75N92019D00031). This work was also supported by grant R01AG063507, R01AG054076, R01AG049607, R01AG059421, R01AG033040, R01AG066524, P30AG066546, U01 AG052409, U01 AG058589 from from the National Institute on Aging and R01 AG017950, UH2/3 NS100605, UF1 NS125513 from National Institute of Neurological Disorders and Stroke and R01HL132320.

Descriptions and Acknowledgments of Replication cohorts

AGES

The Age Gene/Environment Susceptibility (AGES)-Reykjavik study is a prospective longitudinal cohort study of older European white adults who were initially enrolled in the Reykjavik study, established in 1967 (Harris 2007).[16] From 2002 to 2006, 5,764 participants were reexamined for the first wave of the AGES-Reykjavik. Participants underwent a comprehensive assessment which included a clinical examination, questionnaires, a battery of cognitive measures, an MRI scan and a blood draw. Blood samples used for the current analyses were collected at the AGES-Reykjavik study baseline. Serum was prepared using a standardized protocol and stored in 0.5-ml aliquots at −80 °C. Samples were sent to SomaLogic for quantification of 4,137 distinct human proteins targeted by 5,034 SOMAmers on the Novartis SomaScan 5K platform .[17] The AGES-Reykjavik study was approved by the Icelandic Nation Bioethics Committee (VSN 00-063), the Icelandic Data Protection Authority, Iceland and the Institutional Review Board for the National Institute of Aging, National Institutes of Health (NIH), United States. Written informed consent was obtained from all participants. AGES has been funded by NIA contracts N01-AG012100 and HSSN271201200022C, NIH Grant No. 1R01AG065596-01A1, Hjartavernd (the Icelandic Heart Association), and the Althingi (the Icelandic Parliament).

BLSA

The Baltimore Longitudinal Study of Aging (BLSA) is an ongoing longitudinal study in Baltimore, MD designed to assess physical and cognitive measures in a cohort of volunteers recruited from the community (Shock et al., *Normal human aging: The Baltimore longitudinal study of aging, NIH Publication, 1984*). Enrollment began in 1958 with study visits that occurred biannually until 2005, then every 1 to 4 years depending on age (age <60 years, every 4 years; age 60-79 years, every 2 years; age ≥80 years, every year). For a subset of participants enrolled in the BLSA neuroimaging substudy, study visits occurred annually beginning in 1994. Due to BLSA's continuous enrollment, participants entered the study at different times and thus varied with respect to follow-up times. Participants received comprehensive health and functional screening evaluations at each study visit, and were free of major chronic diseases as well as cognitive and functional impairment at the time of enrollment. PC1 was generated from verbal fluency, verbal memory and the Trail Making Test B performance. Verbal fluency was assessed using Verbal Fluency-Letters (F, A, S) and Verbal Fluency-Categories (fruits, animals, vegetables). Verbal memory was assessed using immediate (sum of 5 learning trials) and long-delay free recall from the California Verbal Learning Test. Verbal fluency and verbal memory scores were calculated from averaging z-scores of the individual measures. The completion time of the Trail Making Test B was first natural log–transformed, z-scored, and then sign-inverted so that higher scores reflect better performance. All measures were standardized prior to analyses. Verbal fluency and Trail Making Test B were initiated in the BLSA beginning in 1984, verbal memory in 1993 and DSST in 2005. Proteins were measured with the SOMAmer-based array method (SomaLogic™), specifically the SomaScan® V4.1 platform. Plasma was collected in 2009-2010 using standardized protocols and frozen at -80°C until analysis; a subset of samples was collected at the time of a first PET scan as part of a separate study. Cognitive task data reflected performance documented at the same BLSA study visit as blood sample collection. Samples from participants that did not pass SomaScan QC criteria were

excluded (n = 7). Values were $\log_2$ transformed, those beyond 5 SDs were winzorized and values were standardized. The BLSA protocol was approved by the Institutional Review Board of the National Institute of Environmental Health Science, National Institutes of Health (03AG0325), all participants gave written informed consent prior to participation and deidentified data were used for analyses. A detailed description of the BLSA study design has been published elsewhere (Shock et al., *Normal human aging: The Baltimore longitudinal study of aging*) along with an explanation of procedures for determining cognitive status (Kawas et al., *Age-specific incidence rates of Alzheimer's disease*). M. R. Duggan, T. Tanaka, J. Candia, K. A. Walker, L. Ferrucci, L.J. Launer, O. Meirelles are funded by the National Institute on Aging Intramural Research Program. This study was funded, in part, by the National Institute on Aging Intramural Research Program. We thank the BLSA participants and staff for their participation and continued dedication.

CARDIA

The Coronary Artery Risk Development in Young Adults (CARDIA) study is a prospective multicenter study with 5,115 adult Caucasian and African-American participants aged 18–30 years at recruitment.[18] The recruitment was done from four centers as follows: the total community in Birmingham, AL; selected census tracts in Chicago, IL, and Minneapolis, MN; and from the Kaiser Permanente health plan membership in Oakland, CA. Details of the CARDIA study design have been previously published (1, 2). Nighttime examinations were completed beginning with the study initiation in 1985–1986 and in follow-up years 0, 2, 5, 7, 10, 15, 20, 25, and 30.
The Coronary Artery Risk Development in Young Adults Study (CARDIA) is supported by contracts HHSN268201800003I, HHSN268201800004I, HHSN268201800005I, HHSN268201800006I, and HHSN268201800007I from the National Heart, Lung, and Blood Institute (NHLBI).

LBC1921

The Lothian Birth Cohort 1921 (LBC1921) consists of 550 individuals, most of whom took part in the Scottish Mental Survey of 1932 at the age of ~11 years old.[19] In the survey, they took a validated test of cognitive ability, the MHT version 12. They were recruited to a study to determine influences on cognitive ageing at age ~79 years and have taken part in five waves of testing in later life (at ages 79, 83, 87, 90 and 92 years).[20] For this study, cognitive tests were performed, and plasma was extracted from blood collected in citrate tubes at a mean age of 86.6 years (SD 0.4).

Cognitive tests included Raven's Standard Progressive Matrices, letter-number sequencing and digit symbol coding. From these three cognitive tests, a general cognitive component was derived. The scores from the first unrotated component of a principal component analysis were extracted and labelled as general cognitive function. This component explained 68% of the variance, with individual test loadings ranging from 0.53 to 0.60.

The 92 neurology-related protein biomarkers were measured in plasma by the Proximity Extension Assay technique using the Proseek Multiplex Neurology I 96 × 96 reagents kit by Olink® Proteomics. The data were pre-processed by Olink® using NPX Manager software.

The LBC1921 was supported by the UK's Biotechnology and Biological Sciences Research Council (BBSRC), The Royal Society, and The Chief Scientist Office of the Scottish Government. Genotyping was funded by the BBSRC (BB/F019394/1).

LBC1936

The Lothian Birth Cohort 1936 (LBC1936) consists of 1091 individuals, most of whom took part in the Scottish Mental Survey of 1947 at the age of ~11 years old.[19] In the survey, they took a validated test of cognitive ability, the Moray House Test (MHT) version 12. They were recruited to a study to determine influences on cognitive ageing at age ~70 years and have taken part in six waves of testing in later life (at mean ages 70, 73, 76, 79, 82 and 86 years). At each wave they underwent a series of cognitive and physical tests, with concomitant brain MRI introduced at age ~73 years. For this study, cognitive tests were performed, and plasma was extracted from blood collected in citrate tubes at mean ages of 69.5 (SD 0.8) (for the Olink Inflammation panel) and 72.5 (SD 0.7) (for the Olink Neurology panel) years. The cognitive tests included here were six of the non-verbal subtests from the Wechsler Adult Intelligence Scale-IIIUK (WAIS-III): matrix reasoning, letter-number sequencing, block design, symbol search, digit symbol coding and digit span backwards. From these six cognitive tests, a general cognitive function was derived. The scores from the first unrotated component of a principal component analysis were extracted and labelled as general cognitive function. This component explained 51-52% of the variance, with individual test loadings ranging from 0.37 to 0.44.

The 92 inflammation-related and 92 neurology-related protein biomarkers were measured in plasma by the Proximity Extension Assay technique using the Proseek Multiplex Neurology I 96 × 96 reagents kit by Olink® Proteomics. The data were pre-processed by Olink® using NPX Manager software.

LBC1936 is supported by the Biotechnology and Biological Sciences Research Council, and the Economic and Social Research Council [BB/W008793/1], Age UK (Disconnected Mind project), and the University of Edinburgh. Genotyping was funded by the BBSRC (BB/F019394/1). The Olink® Neurology Proteomics assay was supported by a National Institutes of Health (NIH) research grant R01AG054628.

MESA

The Multi-Ethnic Study of Atherosclerosis (MESA) is a prospective observational cohort comprising 6,814 adults aged 45 to 84 years in 2000 to 2002 who self-reported their race/ethnicity as Non-Hispanic white, Non-Hispanic black, Hispanic, or Chinese. Adults between the ages of 45 and 84 years who were free of clinically apparent cardiovascular disease (CVD) were recruited from six U.S. communities: Baltimore City and Baltimore County, Maryland; Chicago, Illinois; Forsyth County, North Carolina; Los Angeles County, California; Northern Manhattan and the Bronx, New York; and St. Paul, Minnesota. Each field site recruited from locally available sources, which included lists of residents, lists of dwellings, and telephone exchanges. The primary objective of MESA is to determine the characteristics related to the prevalence and progression of subclinical CVD to clinical CVD focusing on age, sex, and race/ethnicity differences in subclinical disease prevalence, risk of progression, and rates of clinical CVD.[21]

Whole genome sequencing (WGS) for the Trans-Omics in Precision Medicine (TOPMed) program was supported by the National Heart, Lung and Blood Institute (NHLBI). WGS

for "NHLBI TOPMed: Multi-Ethnic Study of Atherosclerosis (MESA)" (phs001416.v1.p1) was performed at the Broad Institute of MIT and Harvard (3U54HG003067-13S1). Centralized read mapping and genotype calling, along with variant quality metrics and filtering were provided by the TOPMed Informatics Research Center (3R01HL-117626-02S1). Phenotype harmonization, data management, sample-identity QC, and general study coordination, were provided by the TOPMed Data Coordinating Center (3R01HL-120393-02S1), and TOPMed MESA Multi-Omics (HHSN2682015000031/HSN26800004). The MESA projects are conducted and supported by the National Heart, Lung, and Blood Institute (NHLBI) in collaboration with MESA investigators. Support for the Multi-Ethnic Study of Atherosclerosis (MESA) projects are conducted and supported by the National Heart, Lung, and Blood Institute (NHLBI) in collaboration with MESA investigators. Support for MESA is provided by contracts 75N92020D00001, HHSN268201500003I, N01-HC-95159, 75N92020D00005, N01-HC-95160, 75N92020D00002, N01-HC-95161, 75N92020D00003, N01-HC-95162, 75N92020D00006, N01-HC-95163, 75N92020D00004, N01-HC-95164, 75N92020D00007, N01-HC-95165, N01-HC-95166, N01-HC-95167, N01-HC-95168, N01-HC-95169, UL1-TR-000040, UL1-TR-001079, UL1-TR-001420, UL1TR001881, DK063491, and R01HL105756. The authors thank the other investigators, the staff, and the participants of the MESA study for their valuable contributions. A full list of participating MESA investigators and institutes can be found at http://www.mesa-nhlbi.org.

Rhineland Study

The Rhineland Study is an ongoing, single-center, population-based cohort study among people aged 30 years and above in Bonn, Germany.[22, 23] All individuals living in two pre-defined recruitment areas are invited to participate in the study. The only exclusion criterion is an insufficient command of the German language to provide informed consent. One of the Rhineland Study's primary objectives is to identify determinants and markers of healthy aging, applying a deep-phenotyping approach. At baseline, participants complete an 8-hour in-depth multi-domain phenotypic assessment and various types of biomaterials are collected. Approval to undertake the study was obtained from the ethics committee of the University of Bonn, Medical Faculty. We obtained written informed consent from all participants in accordance with the Declaration of Helsinki.

Stratified random sampling according to age and sex was used to select a subset (n=720) of Rhineland Study baseline participants for proteomic analyses using Olink Explore 3072 panel. Specifically, 120 participants (60 women and 60 men) from each of the age categories 30-39 years, 40-49 years, 50-59 years, 60-69 years, 70-79 years, and 80-89 years, were selected. The Rhineland Study protocol includes an one-hour cognitive test battery, which includes classical cognitive tasks and eye movement measures to assess memory performance, processing speed, executive functions and crystallized intelligence.[22] For this analysis, we selected those cognitive measures that are most comparable to those of the other cohorts, namely trail-making test A (TMT-A) performance as a measure of processing speed, delayed recall performance in the Rey Auditory Verbal Learning and Memory Test (AVLT)[24] as a measure of memory performance and trail-making test B (TMT-B) minus TMT-A performance as a measure of executive function. We excluded participants with missing data on the three cognitive measures and the covariates, as well as participants with stroke or dementia, giving us a sample of 667 participants for data analysis.

The Rhineland Study is funded by the German Center for Neurodegenerative Diseases (DZNE). The work was further partly supported by the German Research Foundation (DFG) under Germany's Excellence Strategy (EXC2151-390873048) and SFB1454 (project number 432325352); the Federal Ministry of Education and Research under the Diet-Body-Brain Competence Cluster in Nutrition Research (grant numbers 01EA1410C and 01EA1809C) and in the framework "PreBeDem - Mit Prävention und Behandlung gegen Demenz" (grant number 01KX2230); and the Helmholtz Association under the Initiative and Networking Fund (grant number RA-285/19) and the 2023 Innovation Pool.

Three City Study (3C)

The 3C-Study is a cohort study conducted in three French cities (Bordeaux, Dijon, and Montpellier), comprising 9,294 participants, designed to estimate the risk of dementia and cognitive impairment attributable to vascular factors.[15] Eligibility criteria included living in the city and being registered on the electoral rolls in 1999, 65 years or older, and not institutionalized. The study protocol was approved by the Ethical Committee of the University Hospital of Kremlin-Bicêtre and each participant signed an informed consent. Data reported in this article were obtained in Dijon (3C-Dijon study), where 4,931 individuals were recruited and examined for the initial visit between March 1999 and March 2001.[16] The overall design of the 3C-Dijon study is detailed elsewhere.[15,17]

Plasma samples were drawn from all participants at their initial examination and during follow-up visits.

Olink proteomic profiling is currently being conducted on baseline samples in 1,100 participants from the 3C-Dijon cohort study who also underwent brain MRI. At the time of this analysis only the first 264 samples were available for analysis. General, medical, and neuropsychological examination data were collected during visits to the subjects' homes.[16] For this analysis, we used the following cognitive tests: Benton visual retention, the Delayed recall and the trail making B. The tests were administered by psychologists trained in psychometric testing.

This project is supported by a grant overseen by the French National Research Agency (ANR) as part of the "Investment for the Future Programme" ANR-18-RHUS-0002. It has received funding from the European Union's Horizon 2020 research and innovation programme under grant agreement No 667375. The project also received funding from the French National Research Agency (ANR) through the VASCOGENE and SHIVA projects and from the Leducq TNE 2012 on small vessel disease (PI A Joutel, M Nelson). Computations were performed on the Bordeaux Bioinformatics Center (CBiB) computer resources, University of Bordeaux. Funding support for additional computer resources has been provided to S.D. by the Fondation Claude Pompidou. The Three City Study: The Three City (3C) Study is conducted under a partnership agreement among the Institut National de la Santé et de la Recherche Médicale (INSERM), the University of Bordeaux, and Sanofi-Aventis. The Fondation pour la Recherche Médicale funded the preparation and initiation of the study. The 3C Study is also supported by the Caisse Nationale Maladie des Travailleurs Salariés, Direction Générale de la Santé, Mutuelle Générale de l'Education Nationale (MGEN), Institut de la Longévité, Conseils Régionaux of Aquitaine and Bourgogne, Fondation de France, and Ministry of Research–INSERM Programme "Cohortes et collections de données biologiques." Ilana Caro received a grant from the EUR digital public health. This PhD program is supported within the framework of the PIA3 (Investment for the future). Project reference 17-EURE-0019.

**Whitehall II**	In 1985 to 1988, all civil servants aged 35 to 55 years based in 20 departments in London, UK, were invited to participate in the Whitehall II cohort study, and 73% (n = 10,308) agreed.19 Blood samples for proteomic analyses were collected from a random subsample of 2274 dementia-free individuals in 1997 to 1999.20 Cognitive performance measurements were conducted at this and four subsequent clinical examinations in 2002 to 2004, 2007 to 2009, 2012 to 2013, and 2015 to 2016. Follow-up started from 1997 to 1999 and ended at death, dementia, or in October 2019.[25]

Supplementary References

1. Kunkle BW*, et al.* Genetic meta-analysis of diagnosed Alzheimer's disease identifies new risk loci and implicates Abeta, tau, immunity and lipid processing. *Nat Genet* **51**, 414-430 (2019).

2. Bellenguez C*, et al.* New insights into the genetic etiology of Alzheimer's disease and related dementias. *Nat Genet* **54**, 412-436 (2022).

3. Jansen IE*, et al.* Genome-wide meta-analysis identifies new loci and functional pathways influencing Alzheimer's disease risk. *Nature genetics* **51**, 404-413 (2019).

4. Davies G*, et al.* Study of 300,486 individuals identifies 148 independent genetic loci influencing general cognitive function. *Nature Communications* **9**, 2098 (2018).

5. Yu Z*, et al.* A bidirectional Mendelian randomization study supports causal effects of kidney function on blood pressure. *Kidney Int* **98**, 708-716 (2020).

6. Wuttke M*, et al.* A catalog of genetic loci associated with kidney function from analyses of a million individuals. *Nat Genet* **51**, 957-972 (2019).

7. Stanzick KJ*, et al.* Discovery and prioritization of variants and genes for kidney function in >1.2 million individuals. *Nature Communications* **12**, 4350 (2021).

8. ARIC. The Atherosclerosis Risk in Communities (ARIC) Study: design and objectives. The ARIC investigators. *Am J Epidemiol* **129**, 687-702 (1989).

9. Wright Jacqueline D*, et al.* The ARIC (Atherosclerosis Risk In Communities) Study. *Journal of the American College of Cardiology* **77**, 2939-2959 (2021).

10. Knopman DS, Ryberg S. A verbal memory test with high predictive accuracy for dementia of the Alzheimer type. *Arch Neurol* **46**, 141-145 (1989).

11. Wechsler D. *Wechsler adult intelligence scale-revised*. Psychological Corporation (1981).

12. Tombaugh TN, Kozak J, Rees L. Normative Data Stratified by Age and Education for Two Measures of Verbal Fluency: FAS and Animal Naming. *Archives of Clinical Neuropsychology* **14**, 167-177 (1999).

13. Dawber TR, Kannel WB. The Framingham study. An epidemiological approach to coronary heart disease. *Circulation* **34**, 553-555 (1966).

14. Feinleib M, Kannel WB, Garrison RJ, McNamara PM, Castelli WP. The Framingham Offspring Study. Design and preliminary data. *Prev Med* **4**, 518-525 (1975).

15. Splansky GL*, et al.* The third generation cohort of the national heart, lung, and blood institute's Framingham Heart Study: Design, recruitment, and initial examination. *American Journal of Epidemiology* **165**, 1328-1335 (2007).

16. Harris TB*, et al.* Age, Gene/Environment Susceptibility-Reykjavik Study: multidisciplinary applied phenomics. *Am J Epidemiol* **165**, 1076-1087 (2007).

17. Emilsson V*, et al.* Co-regulatory networks of human serum proteins link genetics to disease. *Science*, eaaq1327 (2018).

18. Lloyd-Jones DM, Lewis CE, Schreiner PJ, Shikany JM, Sidney S, Reis JP. The Coronary Artery Risk Development In Young Adults (CARDIA) Study: JACC Focus Seminar 8/8. *J Am Coll Cardiol* **78**, 260-277 (2021).

19. Taylor AM, Pattie A, Deary IJ. Cohort Profile Update: The Lothian Birth Cohorts of 1921 and 1936. *International Journal of Epidemiology* **47**, 1042-1042r (2018).

20. Harris SE*, et al.* Neurology-related protein biomarkers are associated with cognitive ability and brain volume in older age. *Nature Communications* **11**, 800 (2020).

21. Bild DE*, et al.* Multi-Ethnic Study of Atherosclerosis: Objectives and Design. *American Journal of Epidemiology* **156**, 871-881 (2002).

22. Coors A, Breteler MMB, Ettinger U. Processing speed, but not working memory or global cognition, is associated with pupil diameter during fixation. *Psychophysiology* **59**, e14089 (2022).

23. Liu D, Aziz NA, Pehlivan G, Breteler MMB. Cardiovascular correlates of epigenetic aging across the adult lifespan: a population-based study. *Geroscience*, (2023).

24. Boenniger MM, Staerk C, Coors A, Huijbers W, Ettinger U, Breteler MMB. Ten German versions of Rey's auditory verbal learning test: Age and sex effects in 4,000 adults of the Rhineland Study. *J Clin Exp Neuropsychol* **43**, 637-653 (2021).

25. Lindbohm JV*, et al.* Plasma proteins, cognitive decline, and 20-year risk of dementia in the Whitehall II and Atherosclerosis Risk in Communities studies. *Alzheimer's & dementia : the journal of the Alzheimer's Association* **18**, 612-624 (2022).

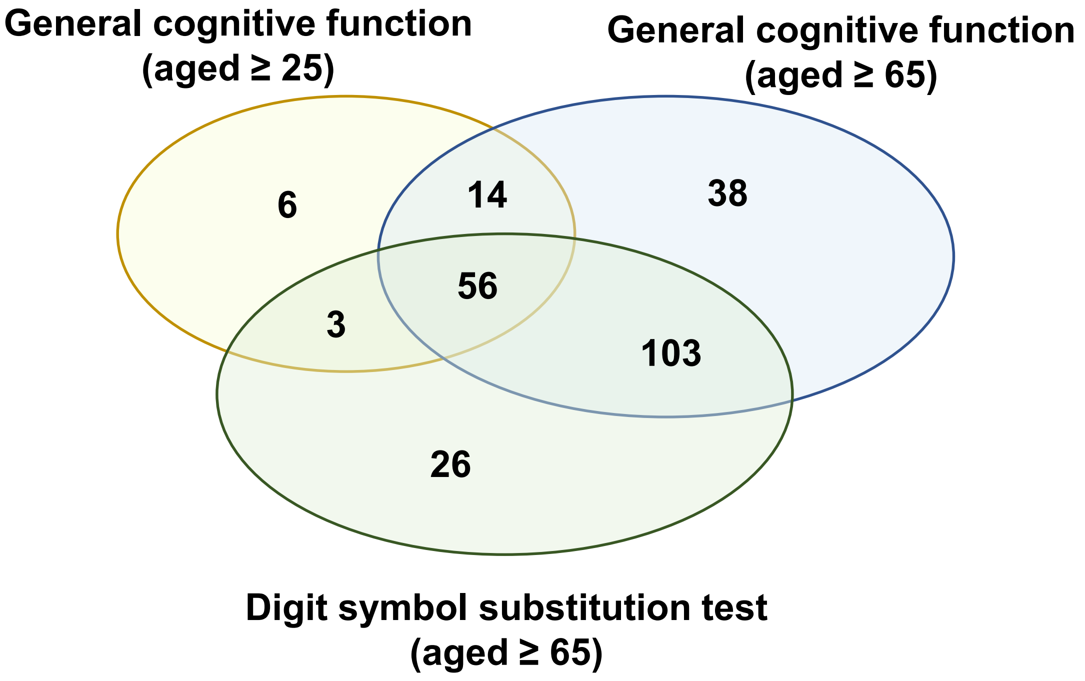

Supplementary Figure 1. Overlaps of significant proteins across the three discovery meta-analyses

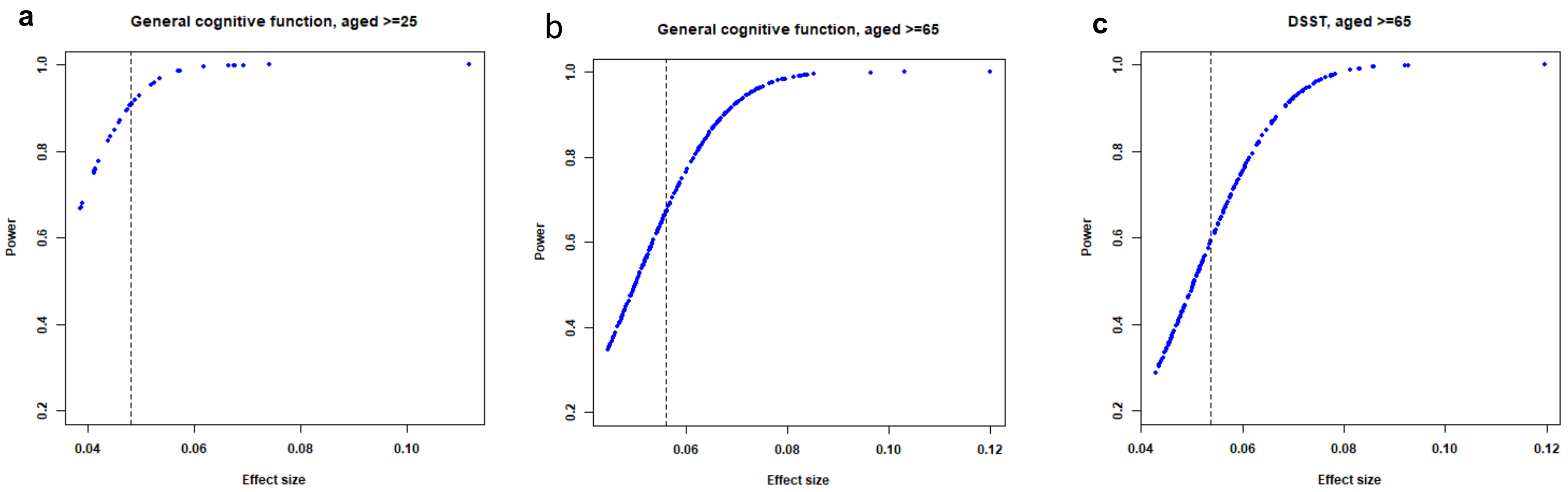

Supplementary Figure 2. Plots showing the power at Bonferroni-corrected significance level for replicating significant proteins of cognitive function based on their effect size from the discovery meta-analysis and the available replication sample sizes on the SomaScan platform: n=8,891 for PC1 of general cognitive function among those aged ≥ 25 and n=5,268 for general cognitive function and the Digit Symbol Substitution Test (DSST) among those aged ≥ 65. The dotted line marks the median of the effect size. The Bonferroni-corrected significance levels for replication were p-value < 0.05/38 = 1.3E-03 for general cognitive function aged ≥ 25 (a), p-value < 0.05/175 = 2.85E-4 for general cognitive function aged ≥ 65 (b), and p-value < 0.05/154 = 3.25E-4 for DSST aged ≥ 65 (c).

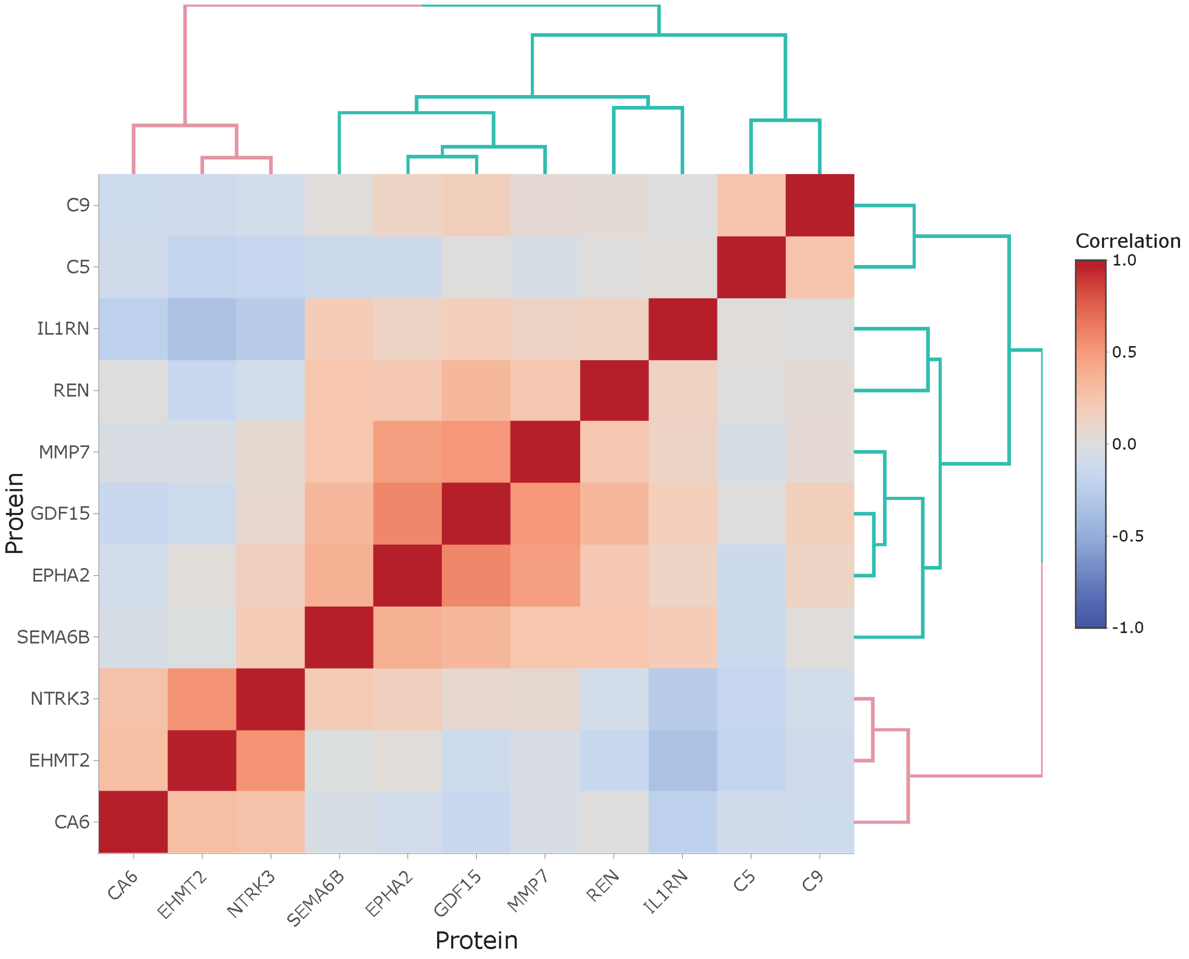

Supplementary figure 3. Plot of the Pearson correlations between proteins that were replicated at Bonferroni-corrected significance using SomaScan data for general cognitive function among those aged ≥ 25 with hierarchical clustering. Median absolute correlation: 0.13 (25th, 75th percentile: 0.06, 0.24).

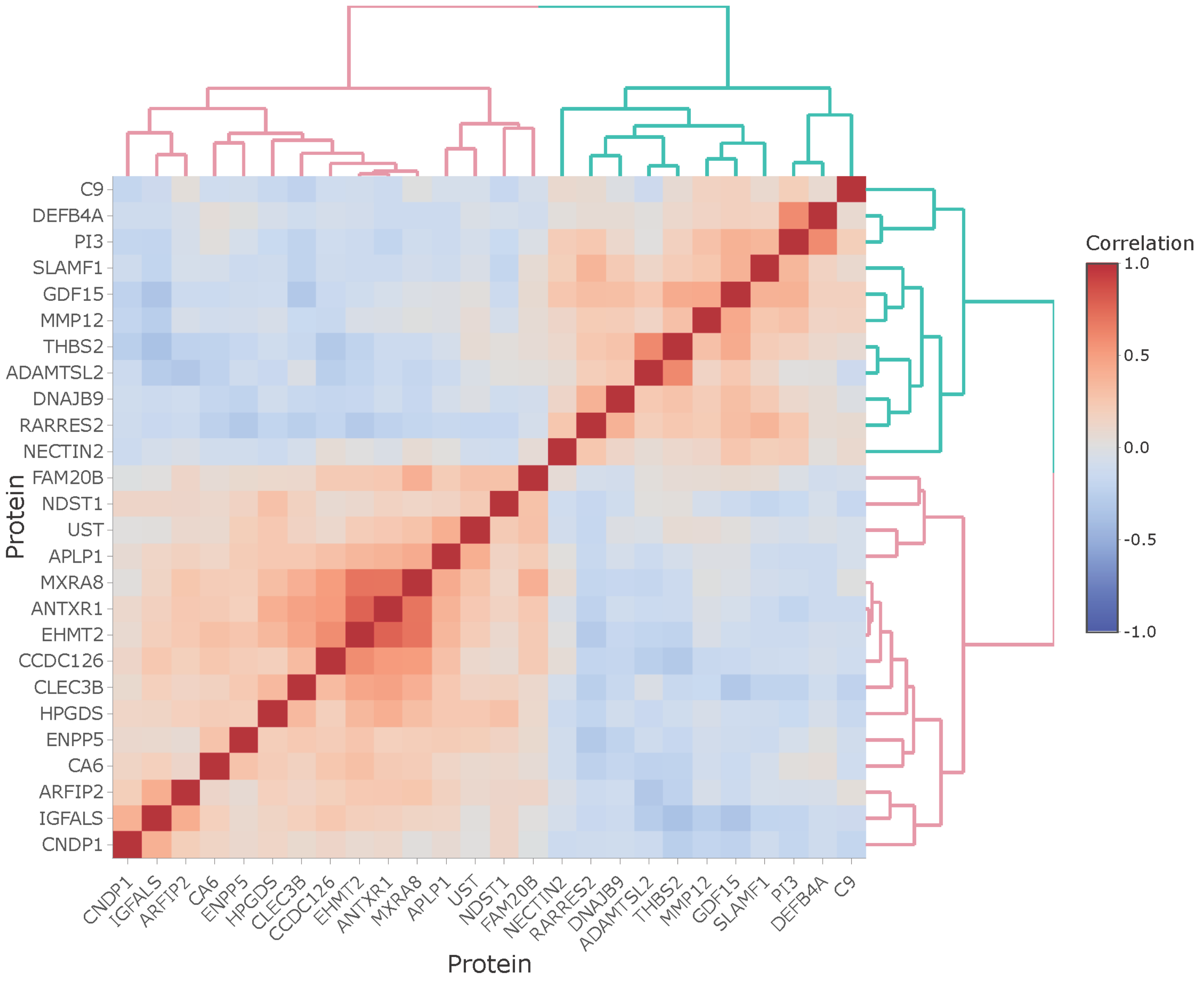

Supplementary figure 4. Plot of the Pearson correlations between proteins that were replicated at Bonferroni-corrected significance using SomaScan data for general cognitive function among those aged ≥ 65 with hierarchical clustering. Median absolute correlation: 0.15 (25th, 75th percentile: 0.09, 0.23).

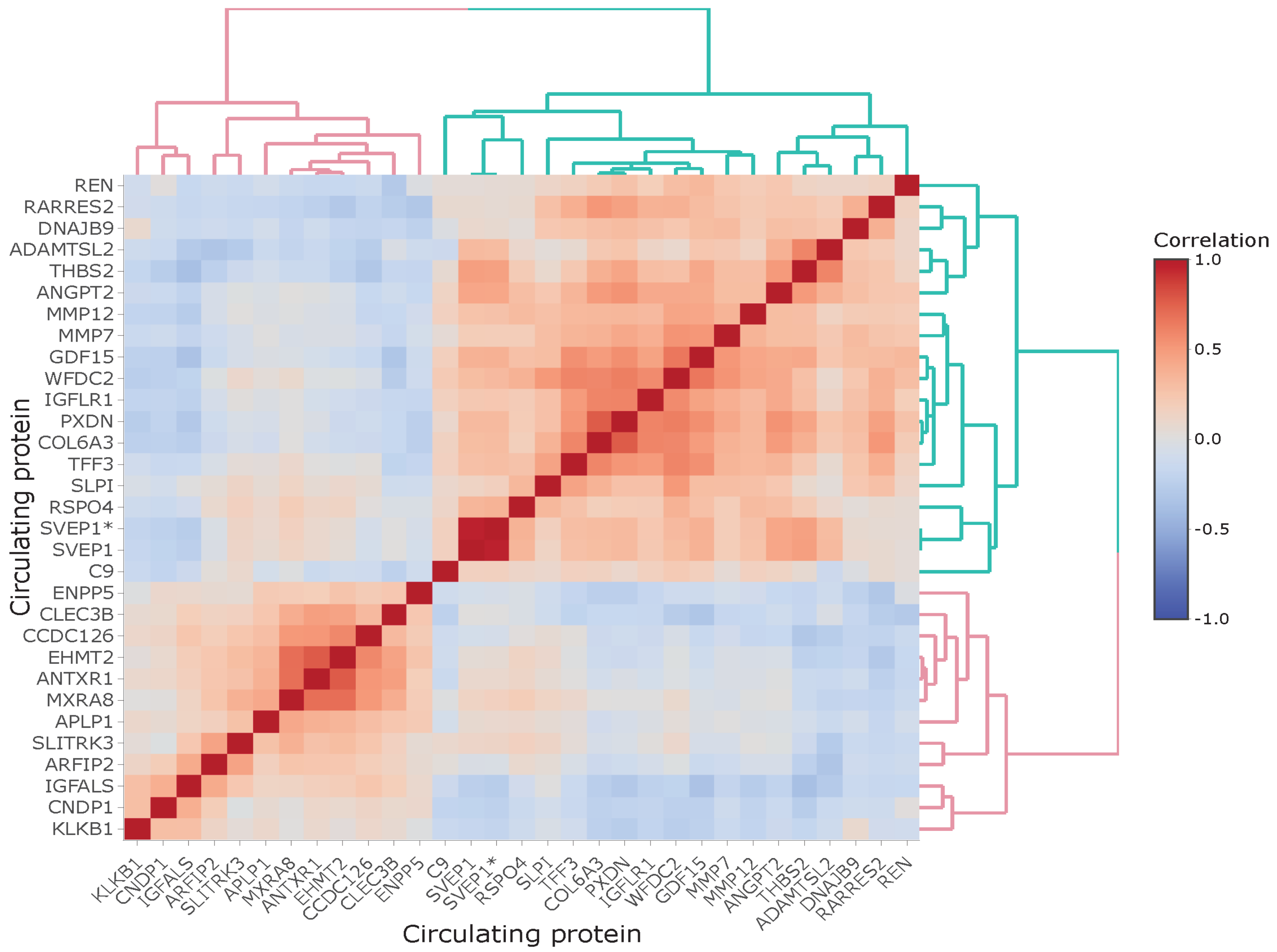

Supplementary figure 5. Plot of the Pearson correlations between 31 proteins that were replicated at Bonferroni-corrected significance using SomaScan data for the Digit Symbol Substitution Test among those aged ≥ 65 with hierarchical clustering. Median absolute correlation: 0.18 (25th, 75th percentile: 0.09, 0.28)

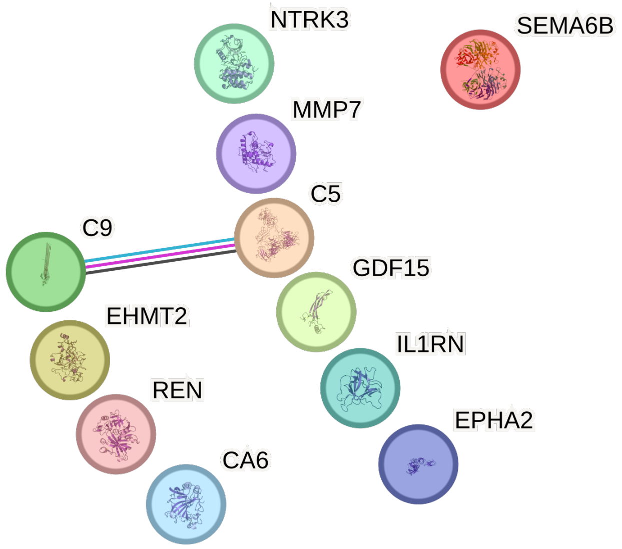

Supplementary figure 6. Potential interactions between 11 proteins that were replicated using SomaScan data at Bonferroni-corrected significance for general cognitive function among those aged >=25. The plot and data were from the STRING database version 12.0 (https://string-db.org/). The edges represent evidence. The sources included were: Experiments, Database, Co-expression, Neighborhood, Gene Fusion, and Co-occurrence. Textmining source was not included.

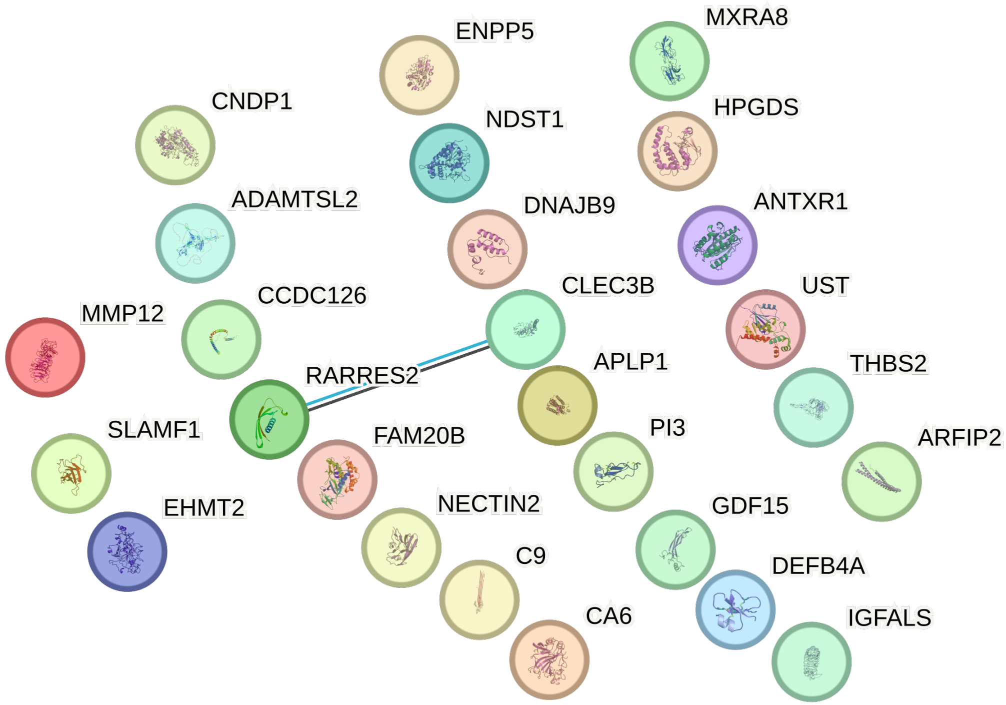

Supplementary figure 7. Potential interactions between 26 proteins that were replicated using SomaScan data at Bonferroni-corrected significance for general cognitive function among those aged >=65. The plot and data were from the STRING database version 12.0 (https://string-db.org/). The edges represent evidence. The sources included were: Experiments, Database, Co-expression, Neighborhood, Gene Fusion, and Co-occurrence. Textmining source was not included.

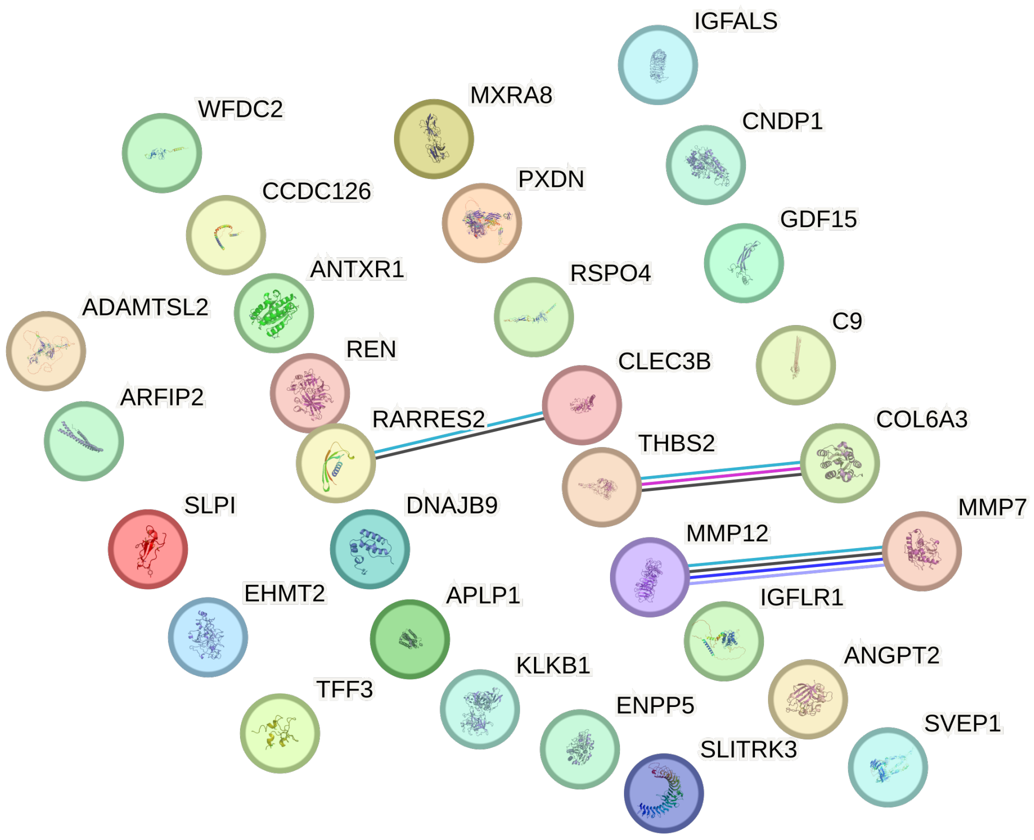

Supplementary figure 8. Potential interactions between 30 unique proteins (31 distinct aptamers) that were replicated using SomaScan data at Bonferroni-corrected significance for the Digit Symbol Substitution Test among those aged ≥ 65. The plot and data were from the STRING database version 12.0 (https://string-db.org/). The edges represent evidence. The sources included were: Experiments, Database, Co-expression, Neighborhood, Gene Fusion, and Co-occurrence. Textmining source was not included. SVEP1 had two distinct aptamers.

Supplementary Figure 9. GO terms that were enriched in the association between circulating proteins and general cognitive function among those aged ≥ 65 in the discovery meta-analysis based on overrepresentation analysis. The proteins on the horizontal axis (n=19) were those significant in the discovery meta-analysis and the significant GO terms. Of these, 13 were replicated: ** indicates those replicated at Bonferroni-corrected significance level (n=2), and * indicates those replicated at FDR < 0.05 only (n=11). The size and color of the circles correspond to –log10(p-value) from the discovery meta-analysis.

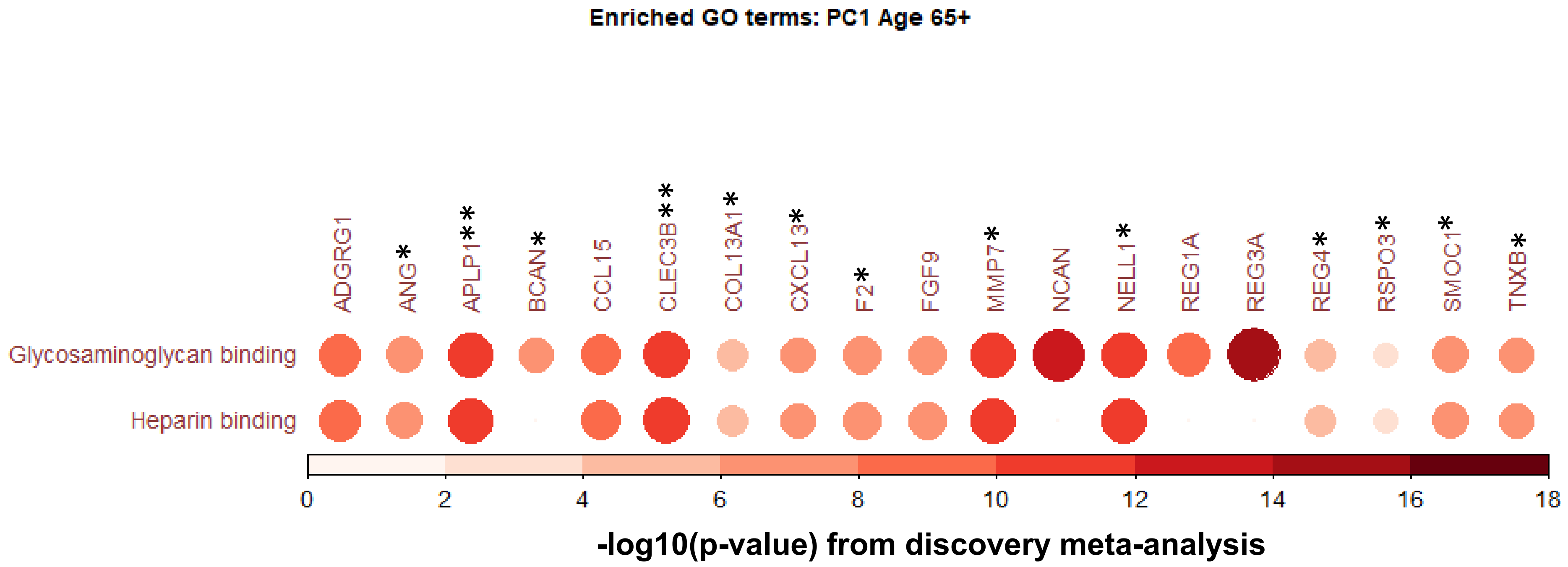

Supplementary Figure 10. GO terms that were enriched in the association between circulating proteins and general cognitive function in discovery meta-analysis with age ≥ 65 based on GSEA. The proteins on the horizontal axis (n=99) were those significant in the discovery meta-analysis and annotated in the significant GO terms. Of these 24 were replicated: ** indicates those replicated at Bonferroni-corrected significance level (n=4), and * indicates those replicated at FDR < 0.05 only (n=20). The size and color of the circles correspond to –log10(p-value) from the discovery meta-analysis.

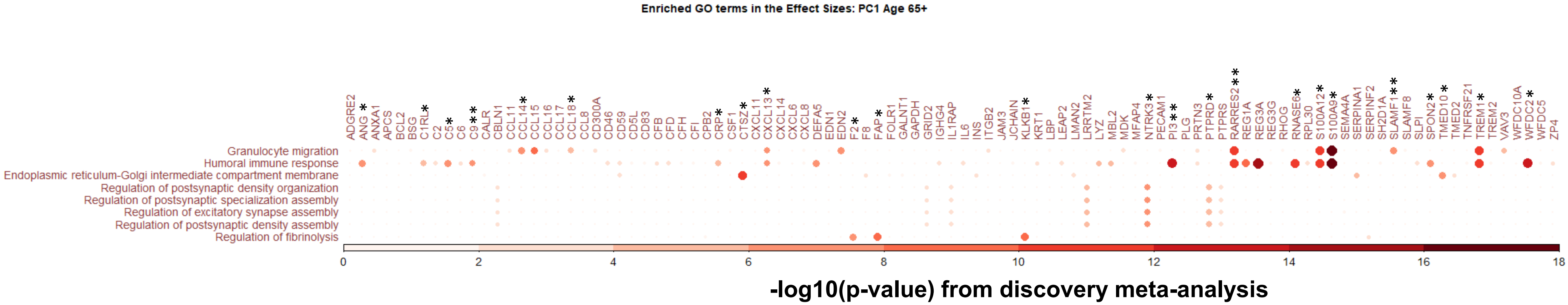

Supplementary Figure 11. GO terms that were enriched in the association between circulating proteins and Digit Symbol Substitution Test among those aged ≥ 65 in the discovery meta-analysis. The proteins on the horizontal axis (n=100) were those significant in the discovery meta-analysis and the significant GO terms. Of these, 22 were replicated: ** indicates those replicated at Bonferroni-corrected significance level (n=7), and * indicates those replicated at FDR < 0.05 only (n=15). The size and color of the circles correspond to –log10(p-value) from the discovery meta-analysis.

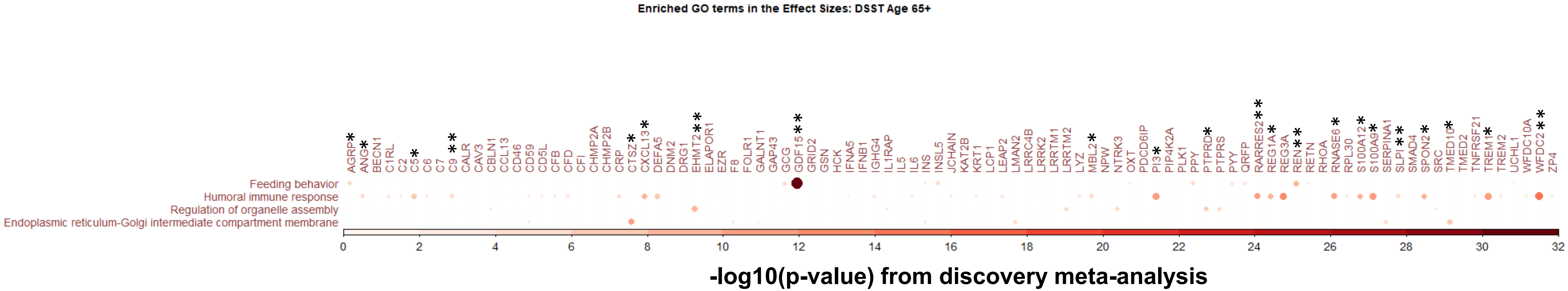

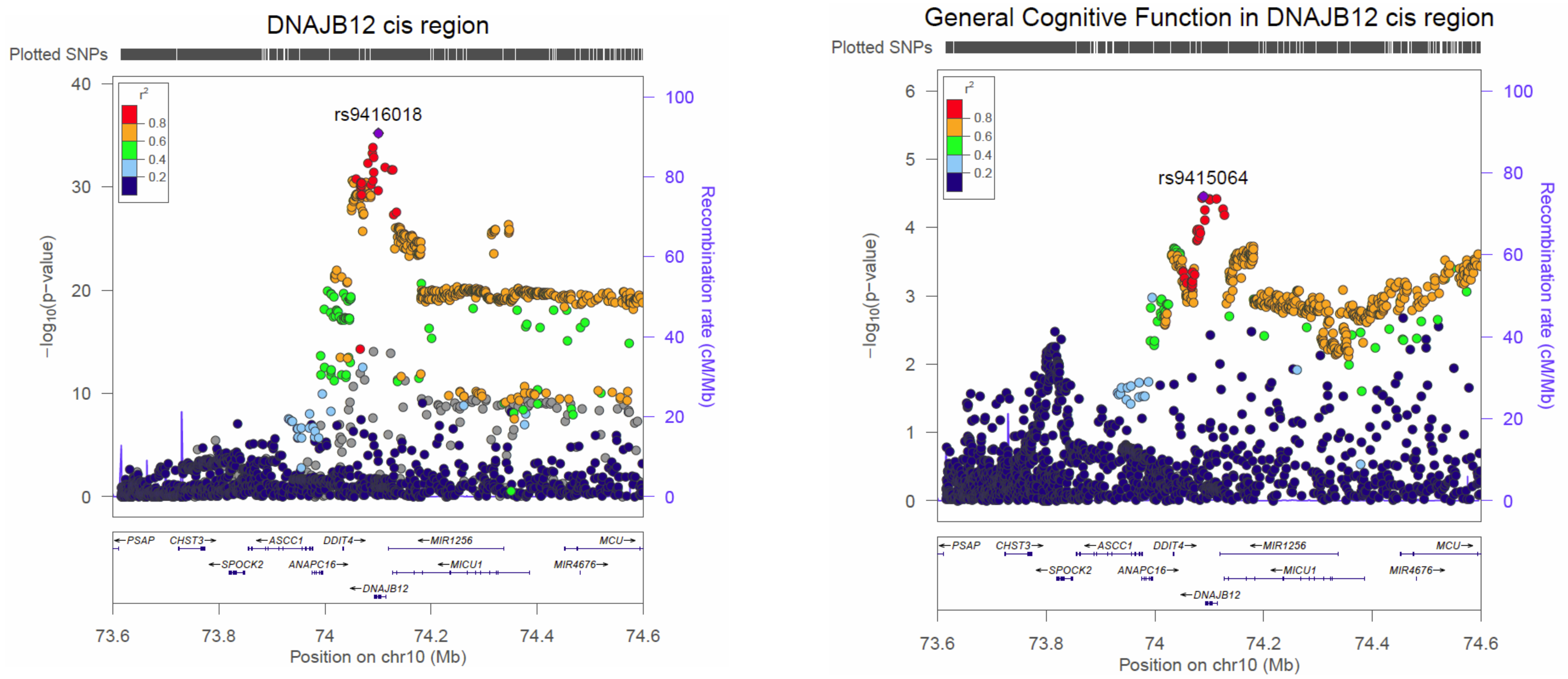

Supplementary figure 12. Plots of the genetic associations at the DNAJB12 promoter region (500kb on both sides, 73.6mb to 74.6mb, b37) for circulating DNAJB12 and general cognitive function used in the colocalization analysis. The datasets were the same as those used for the Mendelian randomization analysis. Genetic associations of circulating DNAJB12 were from a meta-analysis of three pQTL datasets (Sun et al. 2018, Pietzner et al. 2021, and Ferkingstad et al. 2021), and those of general cognitive function were from Davies et al. 2018

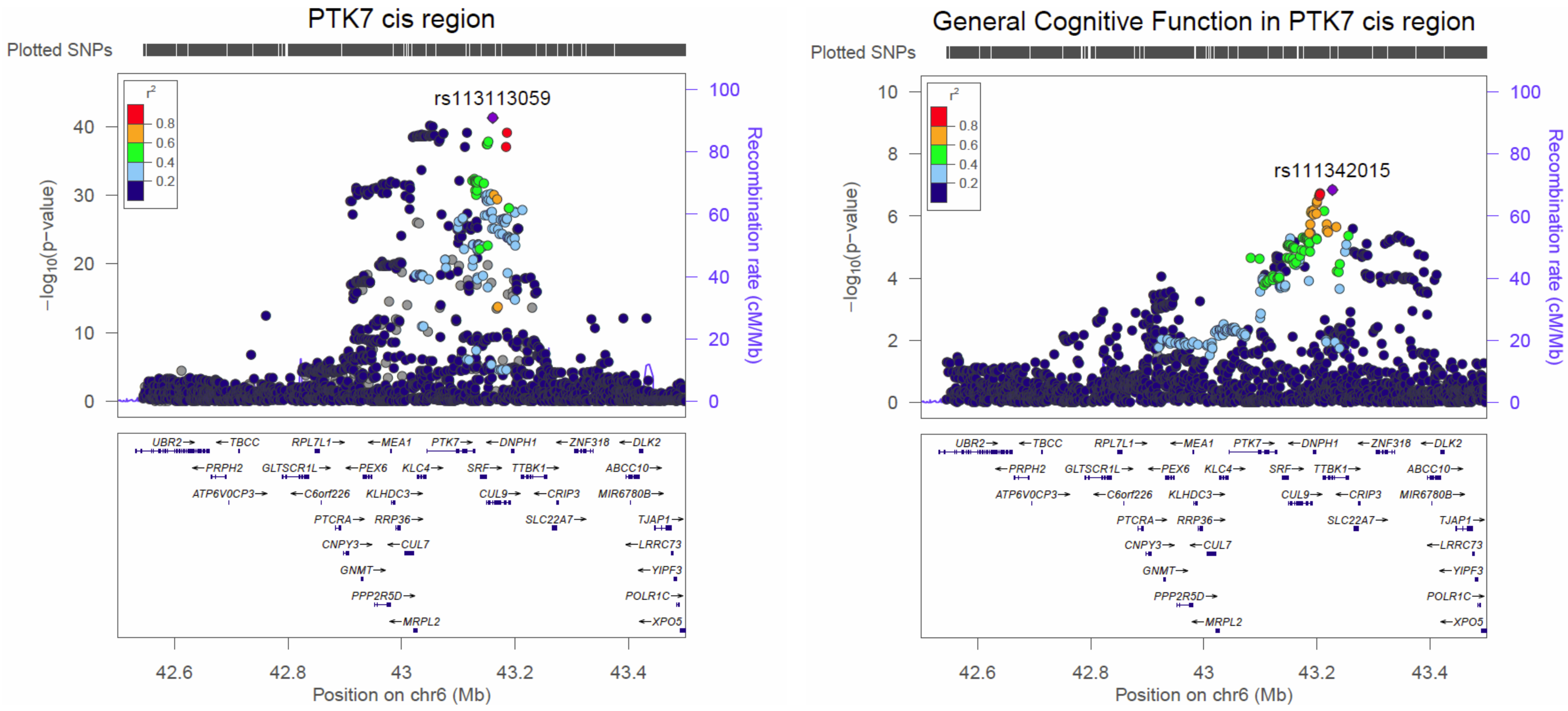

Supplementary figure 13. Plots of the genetic associations at the PTK7 promoter region (500kb on both sides, 42.5mb to 43.5mb, b37) for circulating PTK7 and general cognitive function used in the colocalization analysis. The datasets were the same as those used for the Mendelian randomization analysis. Genetic associations of circulating PTK7 were from a meta-analysis of three pQTL datasets (Sun et al. 2018, Pietzner et al. 2021, and Ferkingstad et al. 2021), and those of general cognitive function were from Davies et al. 2018

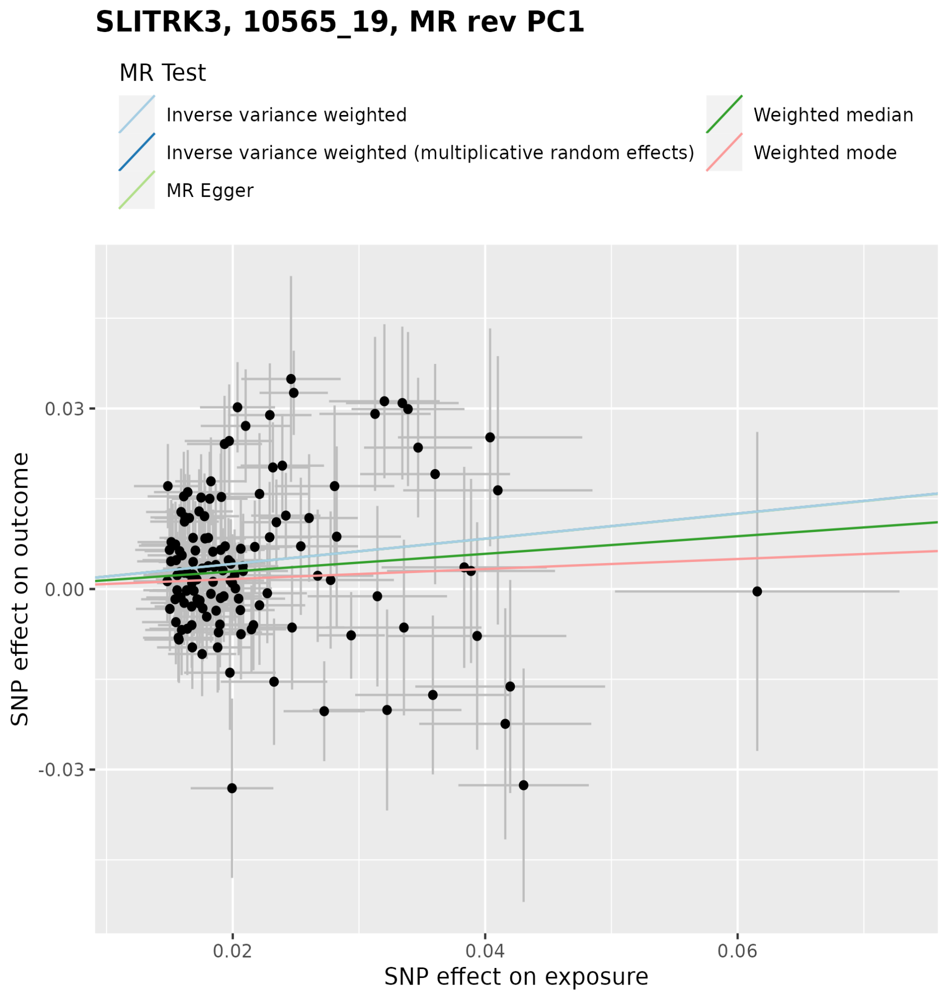

Supplementary figure 14. Scatter plots of the effect sizes of the proxy SNPs of SLITRK3 in two-sample Mendelian randomization analysis with general cognitive function as the exposure and circulating proteins as the outcome.

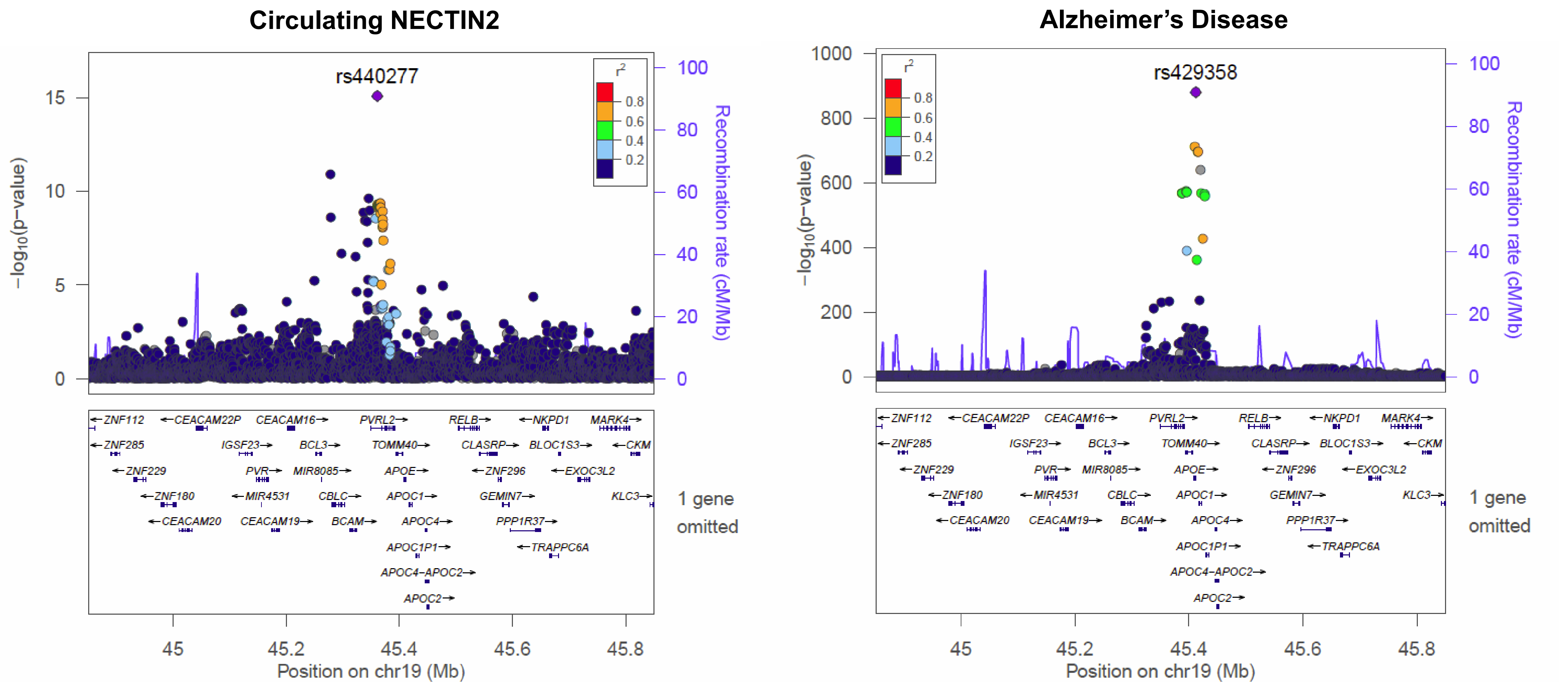

Supplementary figure 15. Plots of the genetic associations at the NECTIN2 promoter region (500kb on both sides, 44.85mb to 45.85mb, b37) for circulating NECTIN2 and Alzheimer's Disease (AD) used in the colocalization analysis. The datasets were the same as those used for the Mendelian randomization analysis. Genetic associations of circulating NECTIN2 were from a meta-analysis of three pQTL datasets (Sun et al. 2018, Pietzner et al. 2021, and Ferkingstad et al. 2021), and those of AD were from stage 1 of Kunkle et al. 2019. Between rs440277 and rs429358, the index SNPs of the two outcomes, the r2 and D' were 0.02 and 0.42, respectively. The linkage disequilibrium measures were from 1000 Genomes Nov 2014 EUR data. The plots were generated using LocusZoom (http://locuszoom.org/genform.php?type=yourdata). NECTIN2 is labeled as PVRL2, an old name of NECTIN2, in the LocusZoom data.

a **All proxy SNPs**

b **Outside APOE region**

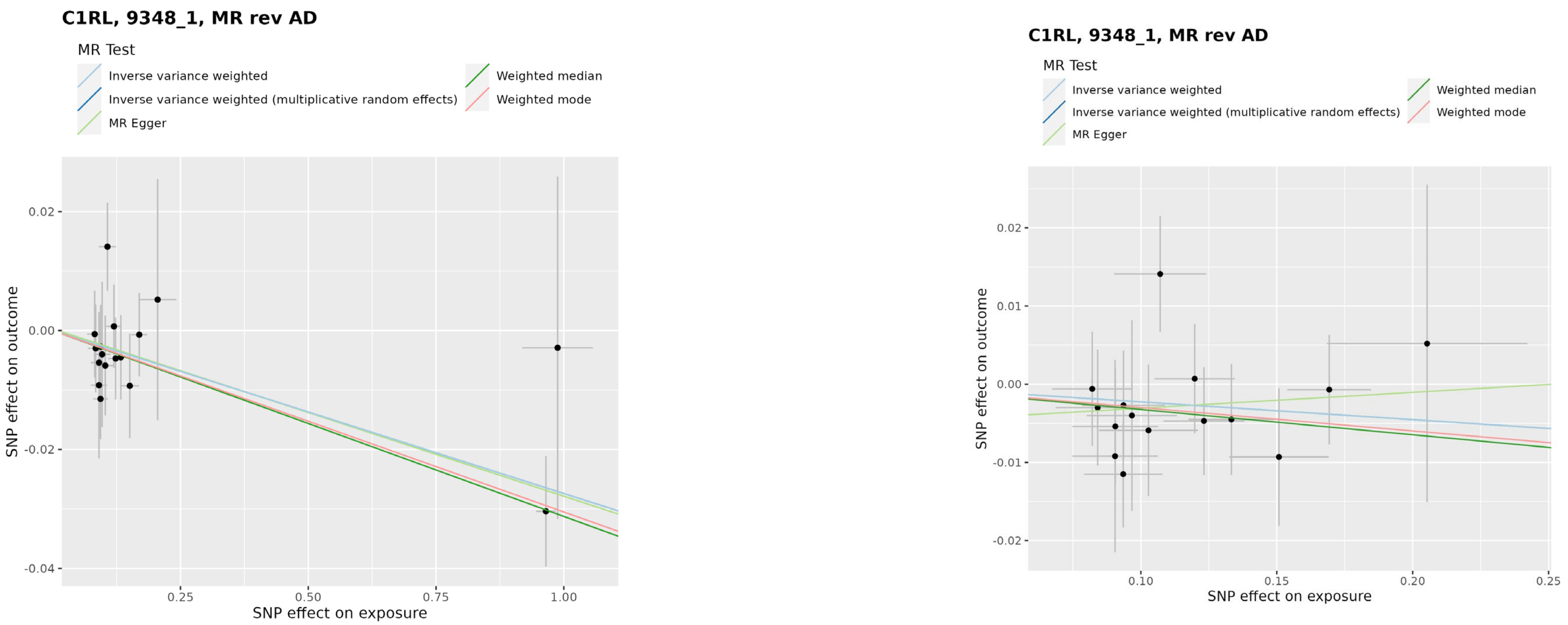

Supplementary figure 16. Scatter plots of proxy SNP effect sizes in the two-sample Mendelian randomization analysis using Alzheimer's disease (AD) susceptibility (Kunkle et al. 2019 stage 1 dataset) as the exposure and circulating C1RL as the outcome. Panel a included all proxy SNPs. Panel b included only those outside of the APOE region. The APOE region is defined as 250 Kb on both sides of the APOE gene. The error bars represent the 95% confidence intervals of the SNP effect sizes on the exposure or the outcome.

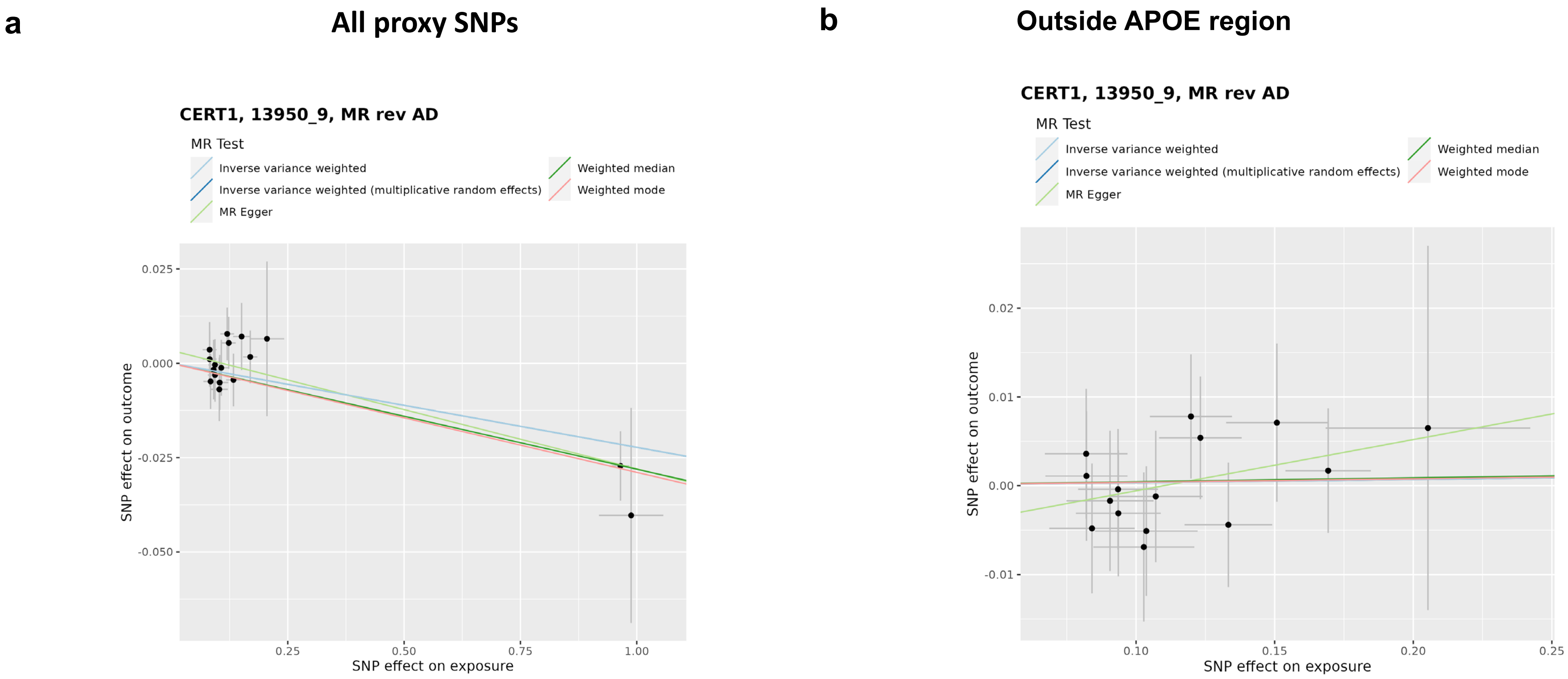

Supplementary figure 17. Scatter plots of proxy SNP effect sizes in the two-sample Mendelian randomization analysis using Alzheimer's disease (AD) susceptibility (Kunkle et al. 2019 stage 1 dataset) as the exposure and circulating CERT1 as the outcome. Panel A included all proxy SNPs. Panel B included only those outside of the APOE region. The APOE region is defined as 250 Kb on both sides of the APOE gene. The error bars represent the 95% confidence intervals of the SNP effect sizes on the exposure or the outcome.

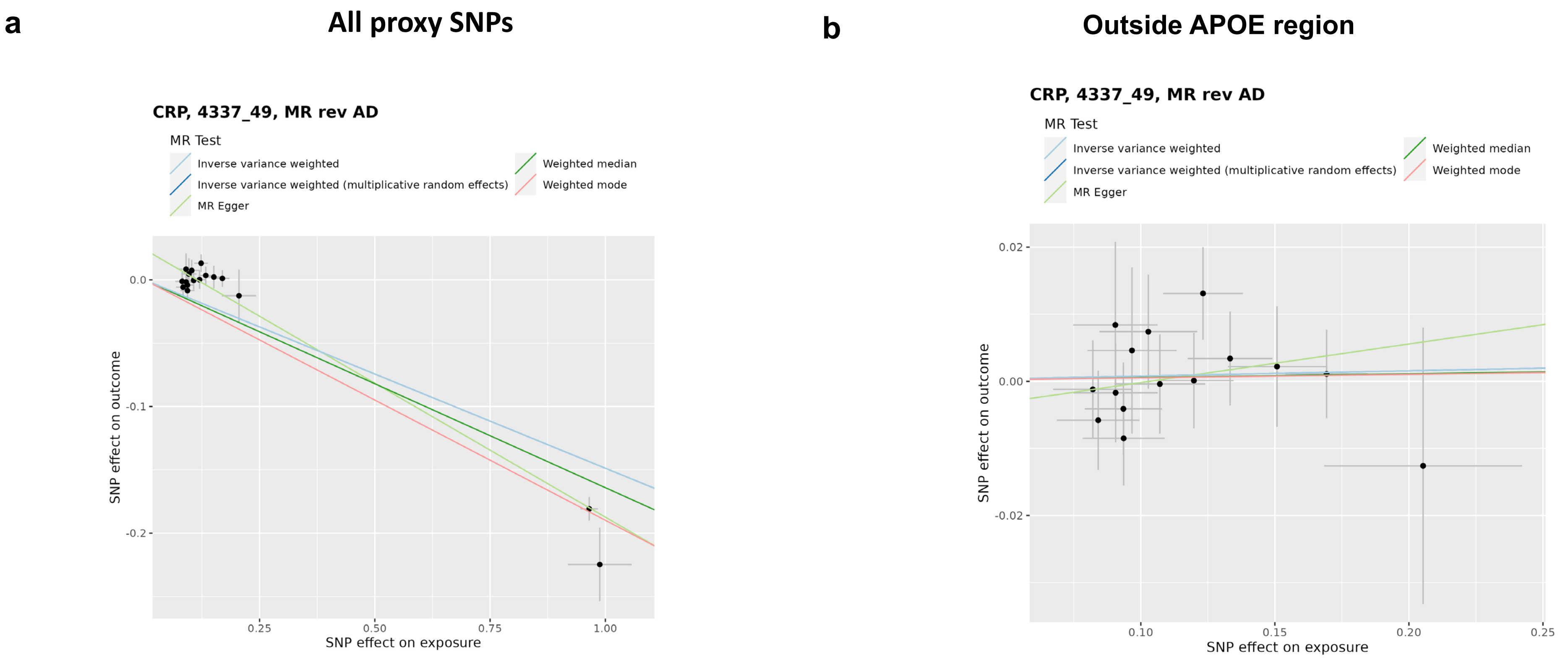

Supplementary figure 18. Scatter plots of proxy SNP effect sizes in the two-sample Mendelian randomization analysis using Alzheimer's disease (AD) susceptibility (Kunkle et al. 2019 stage 1 dataset) as the exposure and circulating CRP as the outcome. Panel A included all proxy SNPs. Panel B included only those outside of the APOE region. The APOE region is defined as 250 Kb on both sides of the APOE gene. The error bars represent the 95% confidence intervals of the SNP effect sizes on the exposure or the outcome.

a **All proxy SNPs**

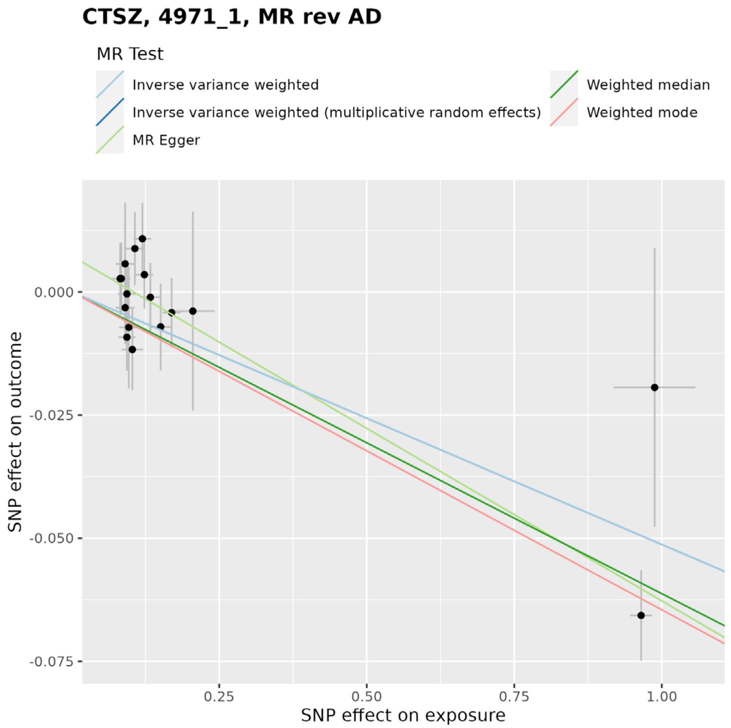

b **Outside APOE region**

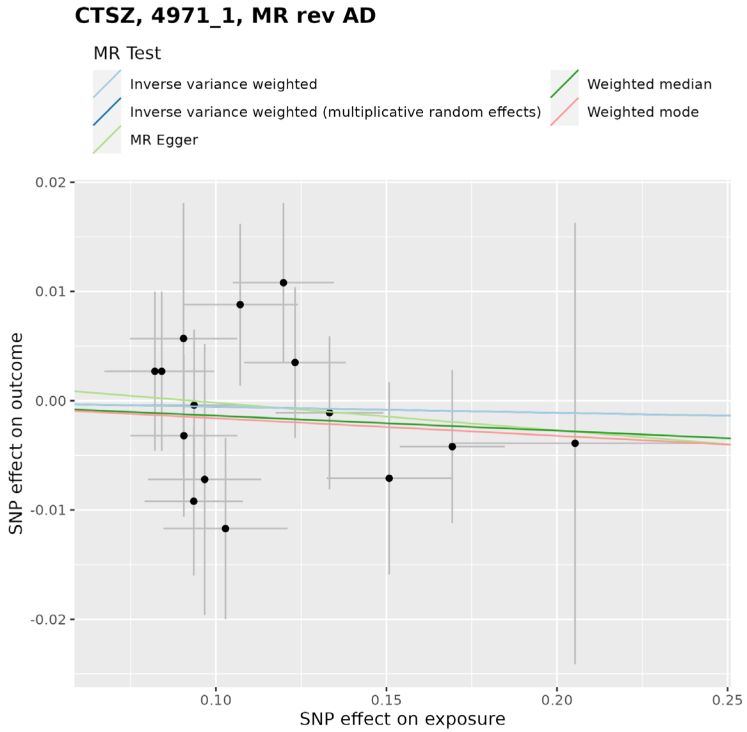

Supplementary figure 19. Scatter plots of proxy SNP effect sizes in the two-sample Mendelian randomization analysis using Alzheimer's disease (AD) susceptibility (Kunkle et al. 2019 stage 1 dataset) as the exposure and circulating CTSZ as the outcome. Panel A included all proxy SNPs. Panel B included only those outside of the APOE region. The APOE region is defined as 250 Kb on both sides of the APOE gene. The error bars represent the 95% confidence intervals of the SNP effect sizes on the exposure or the outcome.

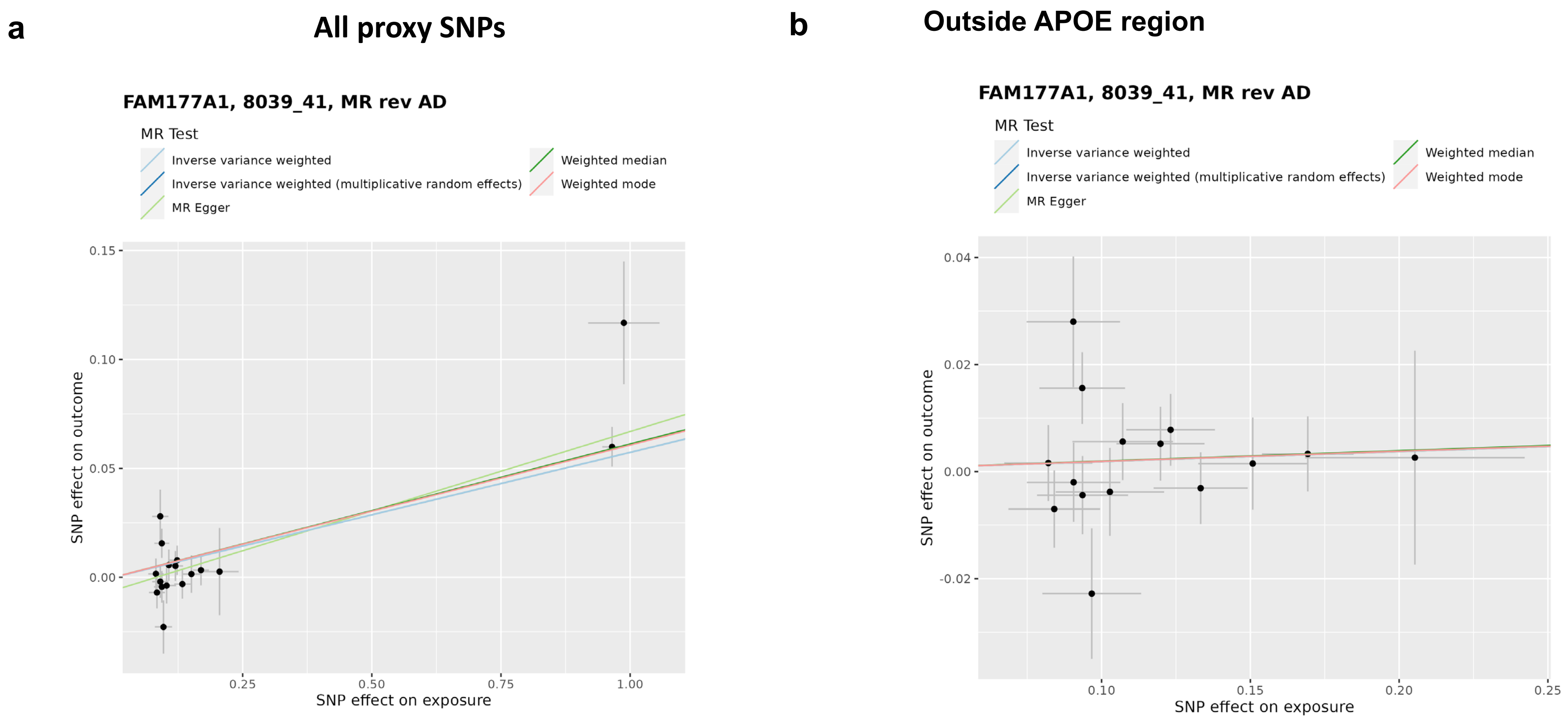

Supplementary figure 20. Scatter plots of proxy SNP effect sizes in the two-sample Mendelian randomization analysis using Alzheimer's disease (AD) susceptibility (Kunkle et al. 2019 stage 1 dataset) as the exposure and circulating FAM177A1 as the outcome. Panel A included all proxy SNPs. Panel B included only those outside of the APOE region. The APOE region is defined as 250 Kb on both sides of the APOE gene. The error bars represent the 95% confidence intervals of the SNP effect sizes on the exposure or the outcome.

a **All proxy SNPs** **b** **Outside APOE region**

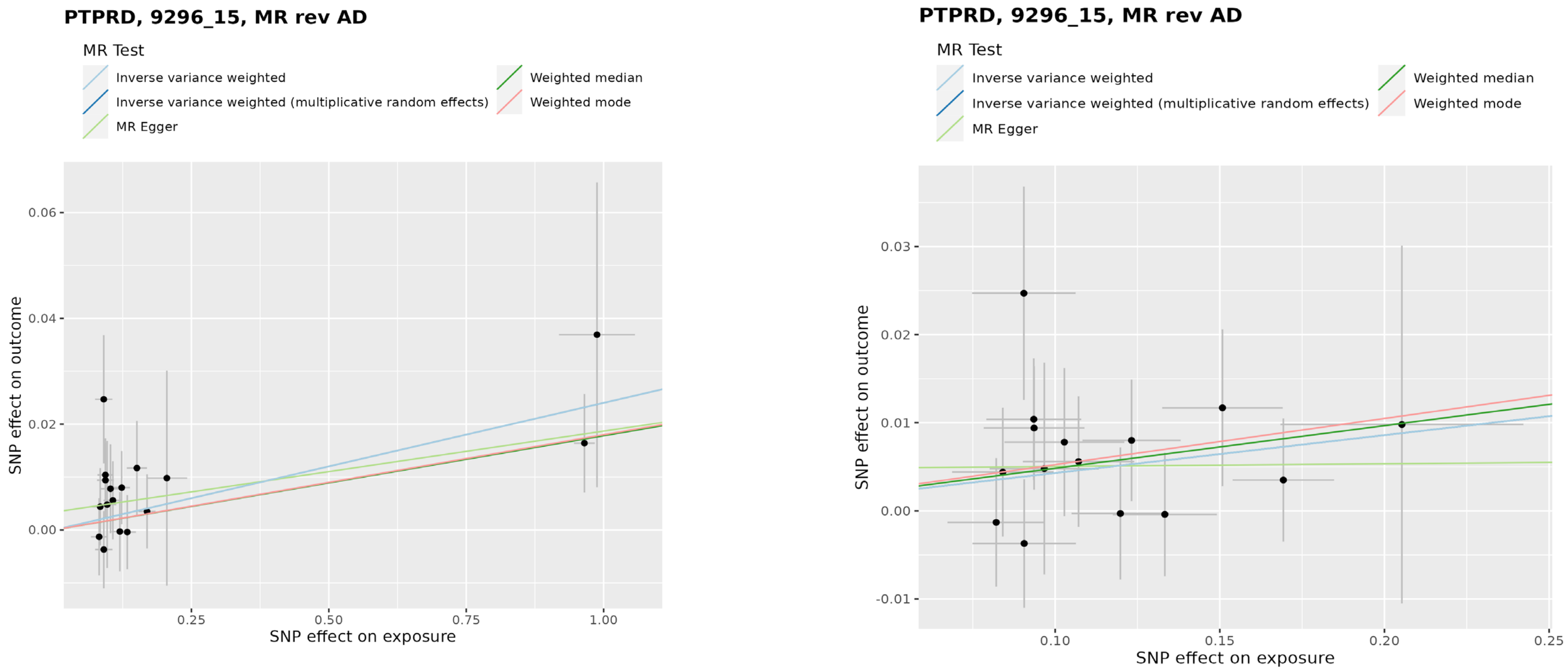

Supplementary figure 21. Scatter plots of proxy SNP effect sizes in the two-sample Mendelian randomization analysis using Alzheimer's disease (AD) susceptibility (Kunkle et al. 2019 stage 1 dataset) as the exposure and circulating PTPRD as the outcome. Panel A included all proxy SNPs. Panel B included only those outside of the APOE region. The APOE region is defined as 250 Kb on both sides of the APOE gene. The error bars represent the 95% confidence intervals of the SNP effect sizes on the exposure or the outcome.

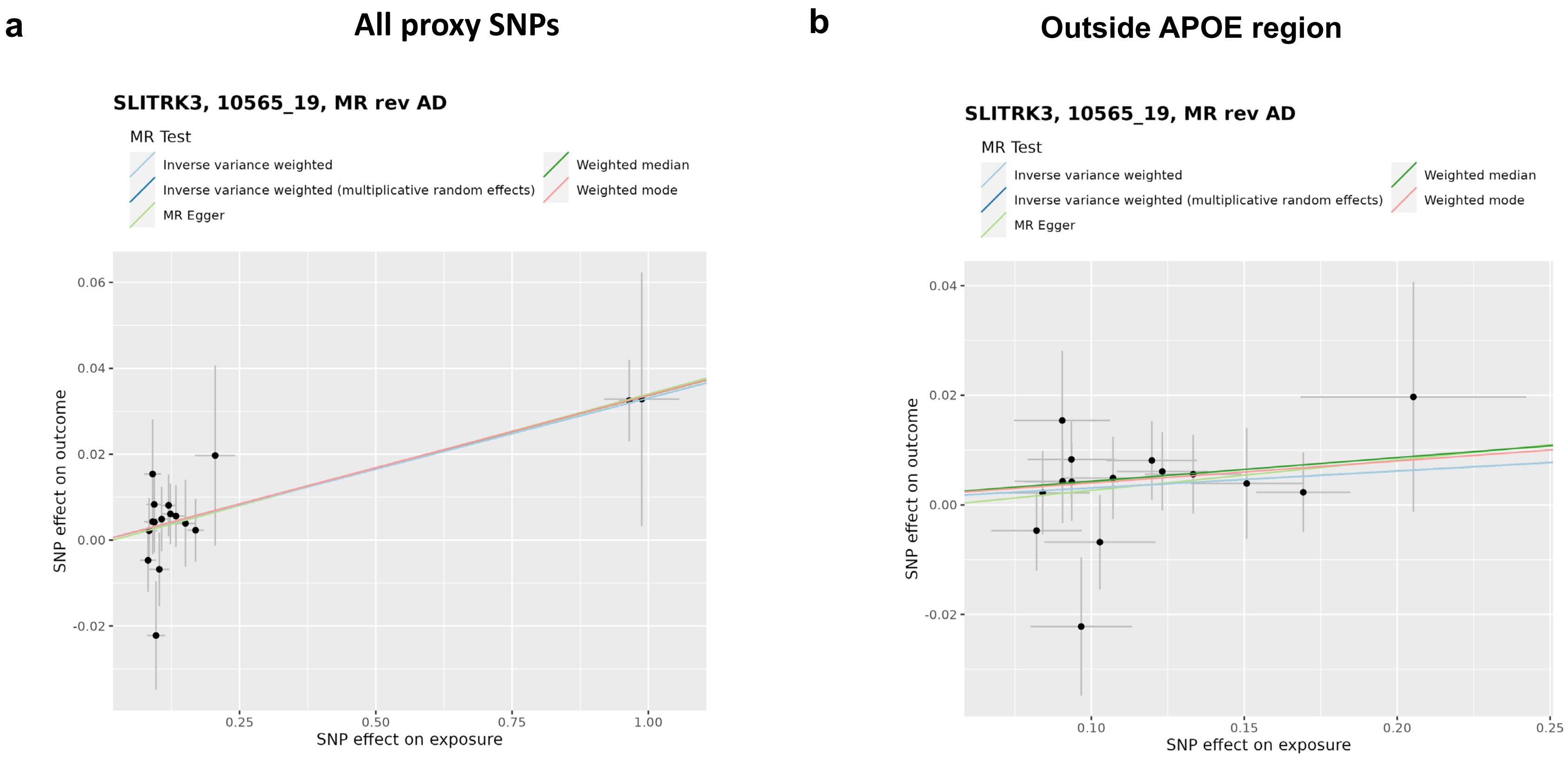

Supplementary figure 22. Scatter plots of proxy SNP effect sizes in the two-sample Mendelian randomization analysis using Alzheimer's disease (AD) susceptibility (Kunkle et al. 2019 stage 1 dataset) as the exposure and circulating SLITRK3 as the outcome. Panel A included all proxy SNPs. Panel B included only those outside of the APOE region. The APOE region is defined as 250 Kb on both sides of the APOE gene. The error bars represent the 95% confidence intervals of the SNP effect sizes on the exposure or the outcome.

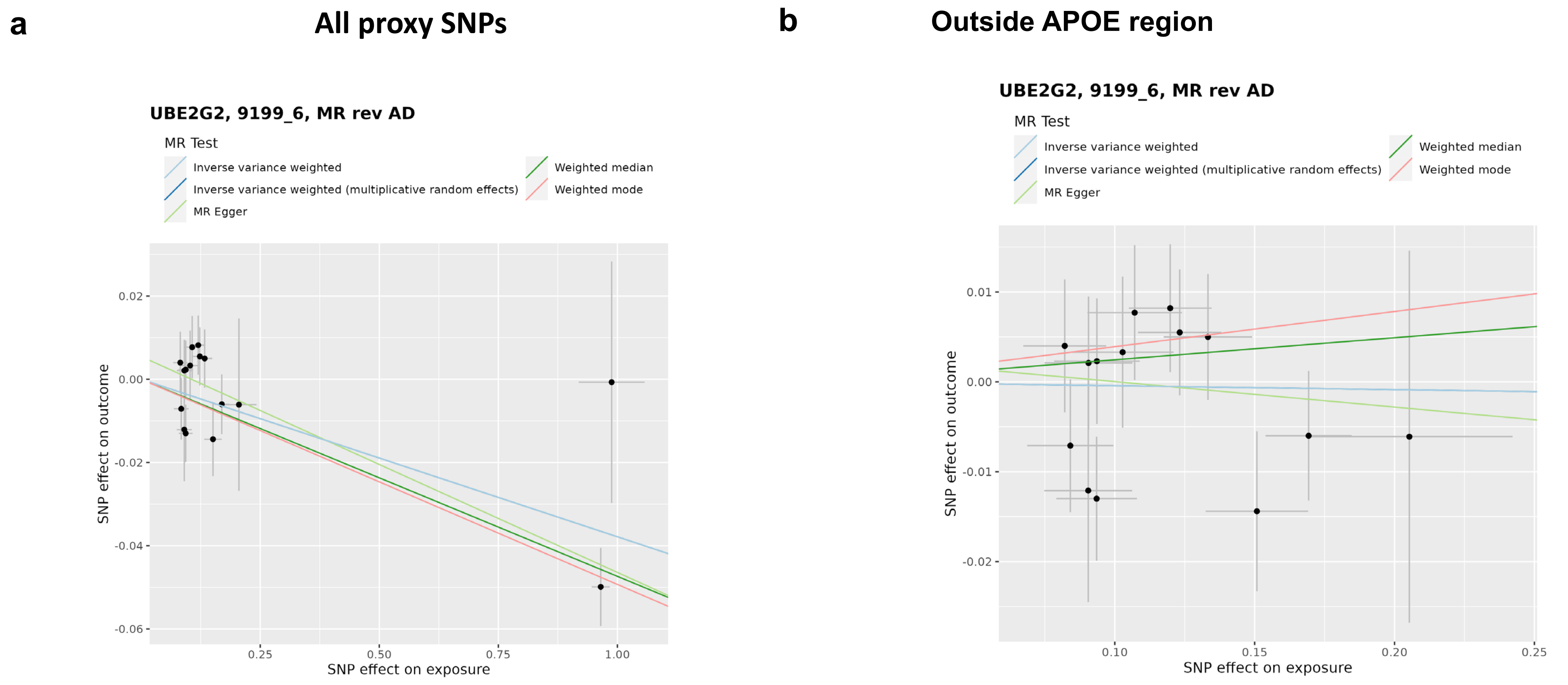

Supplementary figure 23. Scatter plots of proxy SNP effect sizes in the two-sample Mendelian randomization analysis using Alzheimer's disease (AD) susceptibility (Kunkle et al. 2019 stage 1 dataset) as the exposure and circulating SDF2L1 as the outcome. Panel A included all proxy SNPs. Panel B included only those outside of the APOE region. The APOE region is defined as 250 Kb on both sides of the APOE gene. The error bars represent the 95% confidence intervals of the SNP effect sizes on the exposure or the outcome.

a All proxy SNPs

b Outside APOE region

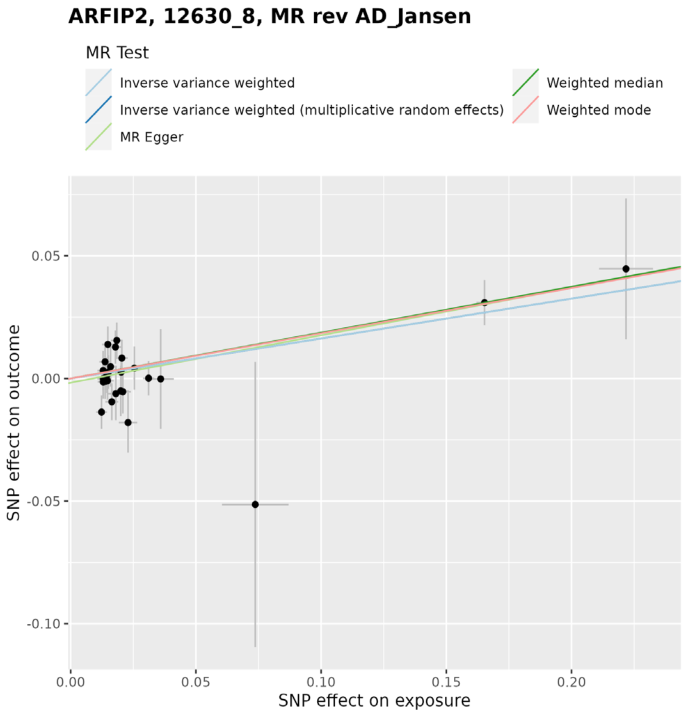

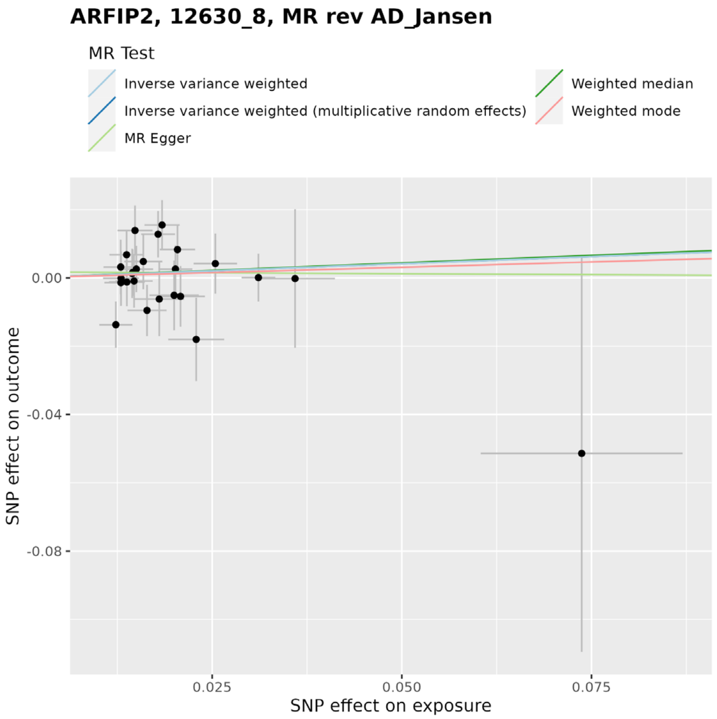

Supplementary figure 24. Scatter plots of proxy SNP effect sizes in the two-sample Mendelian randomization analysis using Alzheimer's disease (AD) susceptibility (Jensen et al. 2019 stage 3 dataset) as the exposure and circulating ARFIP2 as the outcome. Panel a included all proxy SNPs. Panel b included only those outside of the APOE region. The APOE region is defined as 250 Kb on both sides of the APOE gene. The error bars represent the 95% confidence intervals of the SNP effect sizes on the exposure or the outcome.

a **All proxy SNPs** **b** **Outside APOE region**

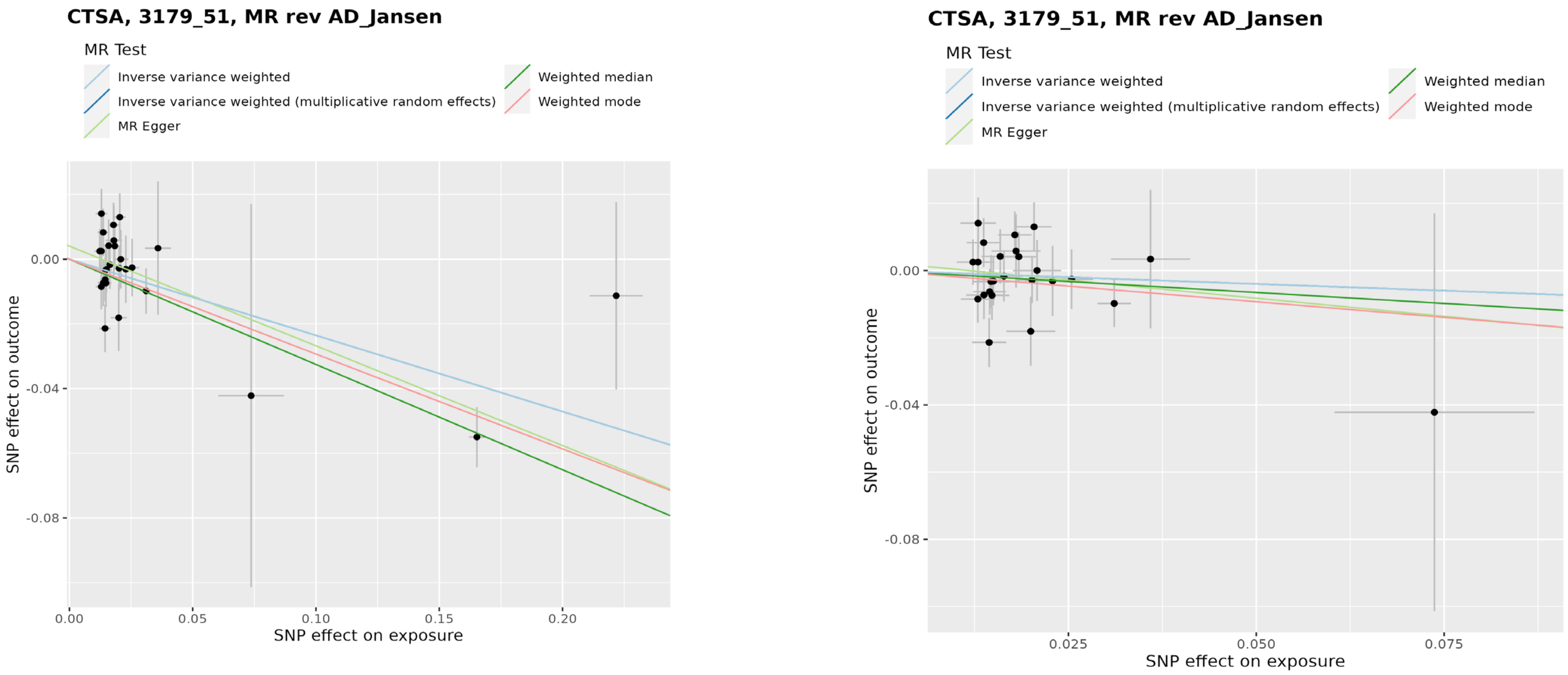

Supplementary figure 25. Scatter plots of proxy SNP effect sizes in the two-sample Mendelian randomization analysis using Alzheimer's disease (AD) susceptibility (Jensen et al. 2019 stage 3 dataset) as the exposure and circulating CTSA as the outcome. Panel a included all proxy SNPs. Panel b included only those outside of the APOE region. The APOE region is defined as 250 Kb on both sides of the APOE gene. The error bars represent the 95% confidence intervals of the SNP effect sizes on the exposure or the outcome.

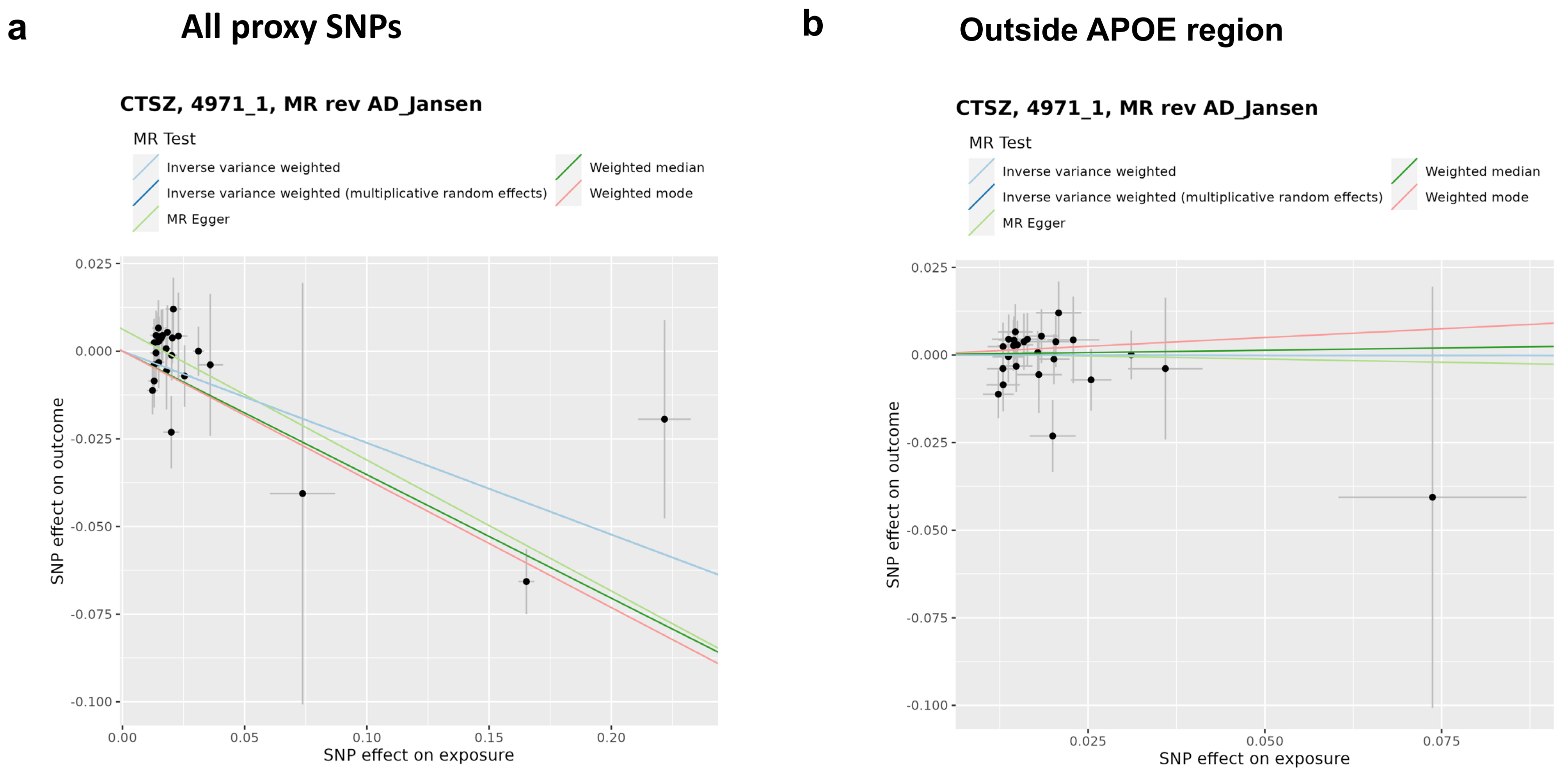

Supplementary figure 26. Scatter plots of proxy SNP effect sizes in the two-sample Mendelian randomization analysis using Alzheimer's disease (AD) susceptibility (Jensen et al. 2019 stage 3 dataset) as the exposure and circulating CTSZ as the outcome. Panel a included all proxy SNPs. Panel b included only those outside of the APOE region. The APOE region is defined as 250 Kb on both sides of the APOE gene. The error bars represent the 95% confidence intervals of the SNP effect sizes on the exposure or the outcome.

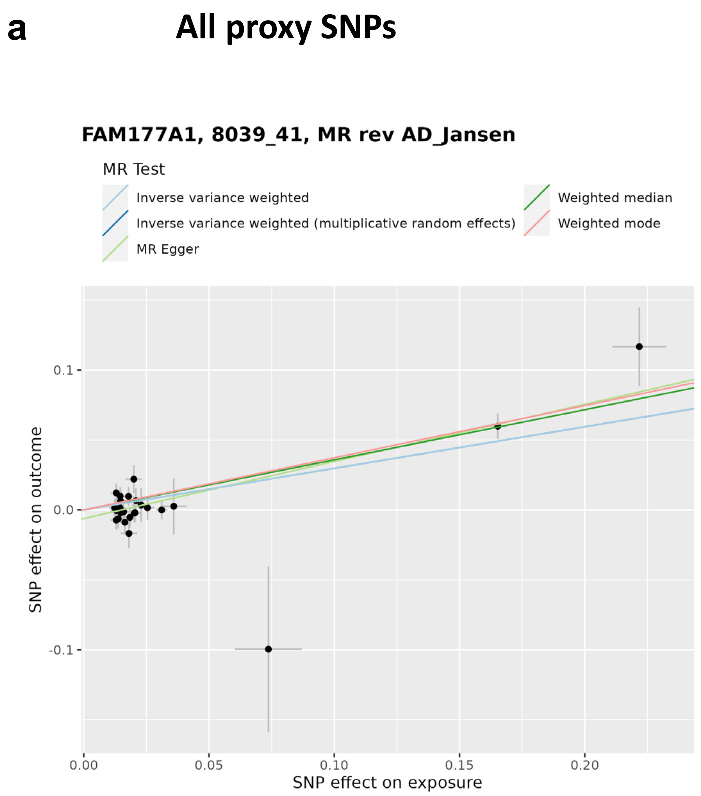

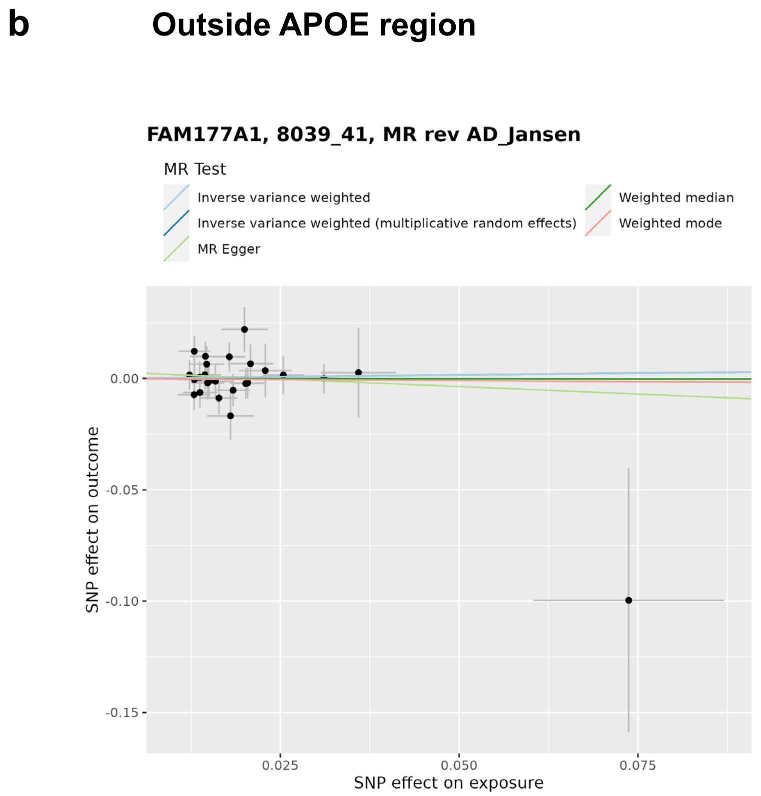

Supplementary figure 27. Scatter plots of proxy SNP effect sizes in the two-sample Mendelian randomization analysis using Alzheimer's disease (AD) susceptibility (Jensen et al. 2019 stage 3 dataset) as the exposure and circulating FAM177A1 as the outcome. Panel a included all proxy SNPs. Panel b included only those outside of the APOE region. The APOE region is defined as 250 Kb on both sides of the APOE gene. The error bars represent the 95% confidence intervals of the SNP effect sizes on the exposure or the outcome.

a All proxy SNPs

b Outside APOE region

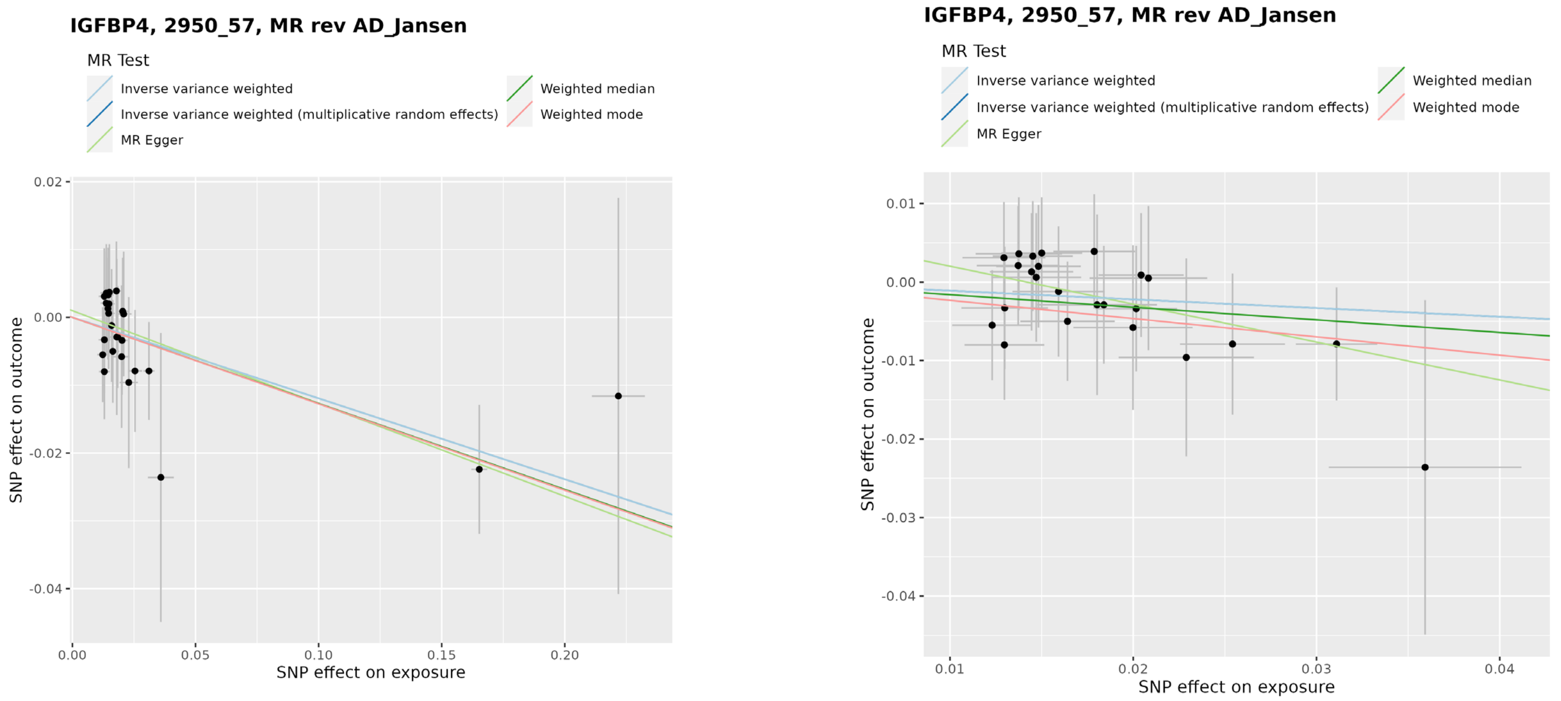

Supplementary figure 28. Scatter plots of proxy SNP effect sizes in the two-sample Mendelian randomization analysis using Alzheimer's disease (AD) susceptibility (Jensen et al. 2019 stage 3 dataset) as the exposure and circulating IGFBP4 as the outcome. Panel a included all proxy SNPs. Panel b included only those outside of the APOE region. The APOE region is defined as 250 Kb on both sides of the APOE gene. The error bars represent the 95% confidence intervals of the SNP effect sizes on the exposure or the outcome.

a **All proxy SNPs** **b** **Outside APOE region**

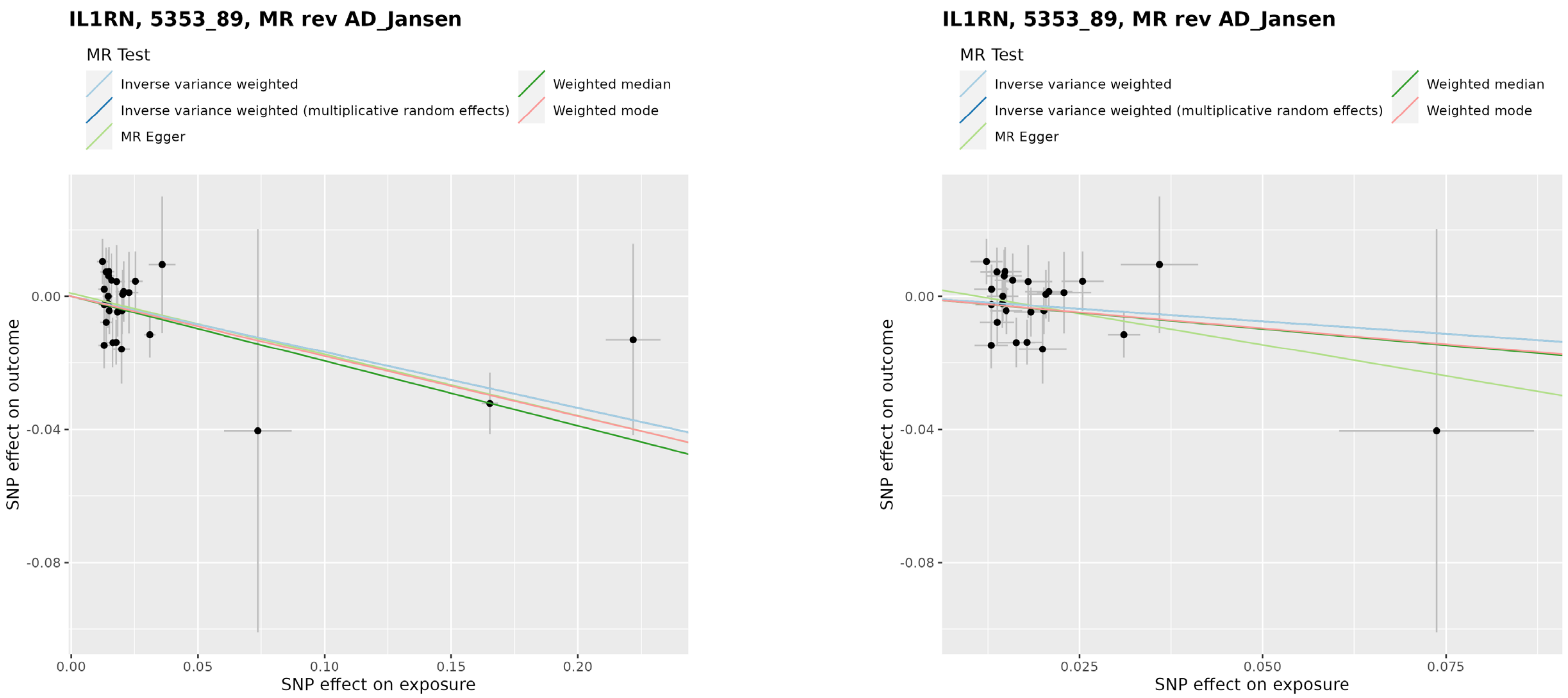

Supplementary figure 29. Scatter plots of proxy SNP effect sizes in the two-sample Mendelian randomization analysis using Alzheimer's disease (AD) susceptibility (Jensen et al. 2019 stage 3 dataset) as the exposure and circulating IL1RN as the outcome. Panel a included all proxy SNPs. Panel b included only those outside of the APOE region. The APOE region is defined as 250 Kb on both sides of the APOE gene. The error bars represent the 95% confidence intervals of the SNP effect sizes on the exposure or the outcome.

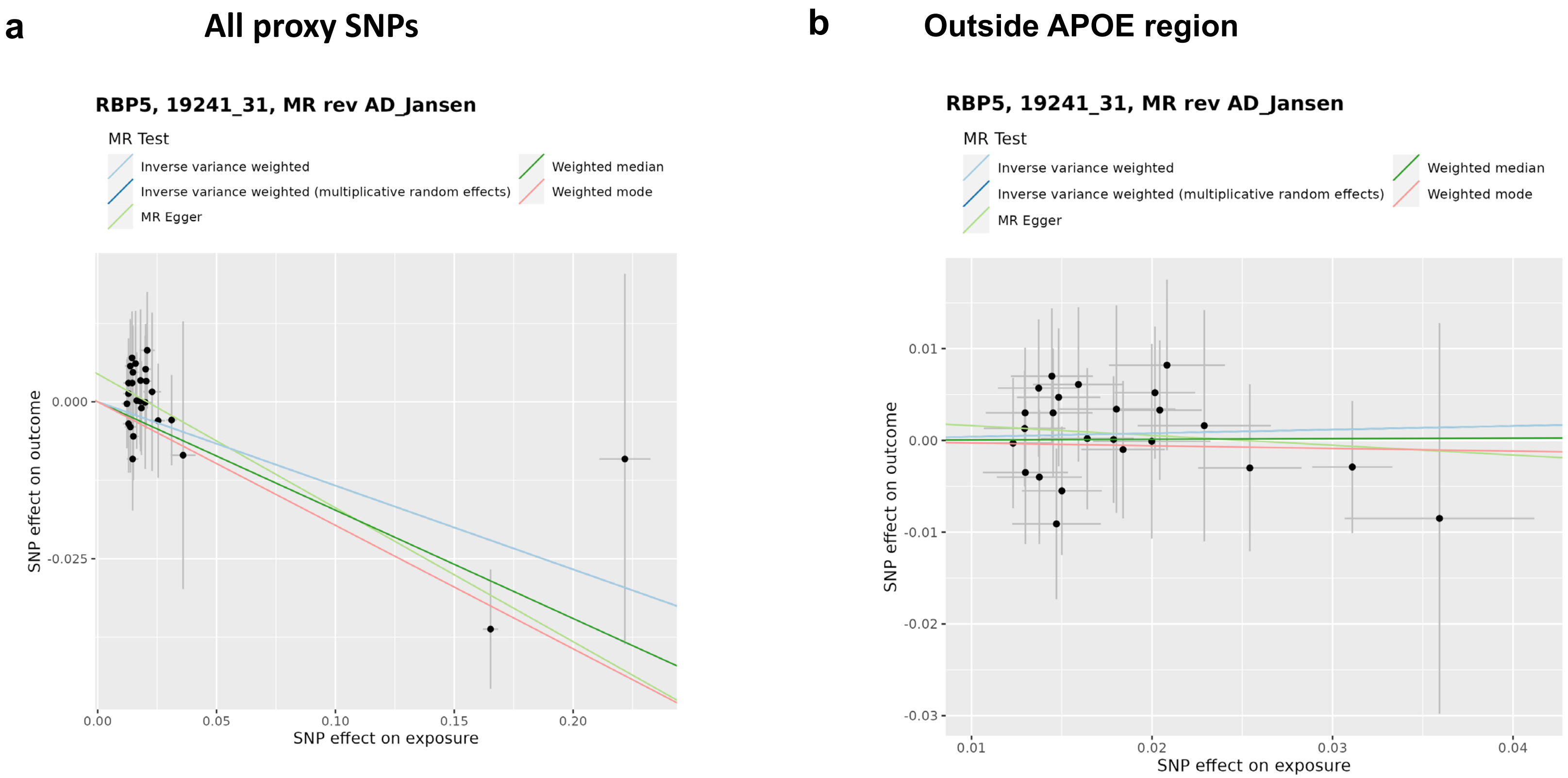

Supplementary figure 30. Scatter plots of proxy SNP effect sizes in the two-sample Mendelian randomization analysis using Alzheimer's disease (AD) susceptibility (Jensen et al. 2019 stage 3 dataset) as the exposure and circulating RPB5 as the outcome. Panel a included all proxy SNPs. Panel b included only those outside of the APOE region. The APOE region is defined as 250 Kb on both sides of the APOE gene. The error bars represent the 95% confidence intervals of the SNP effect sizes on the exposure or the outcome.

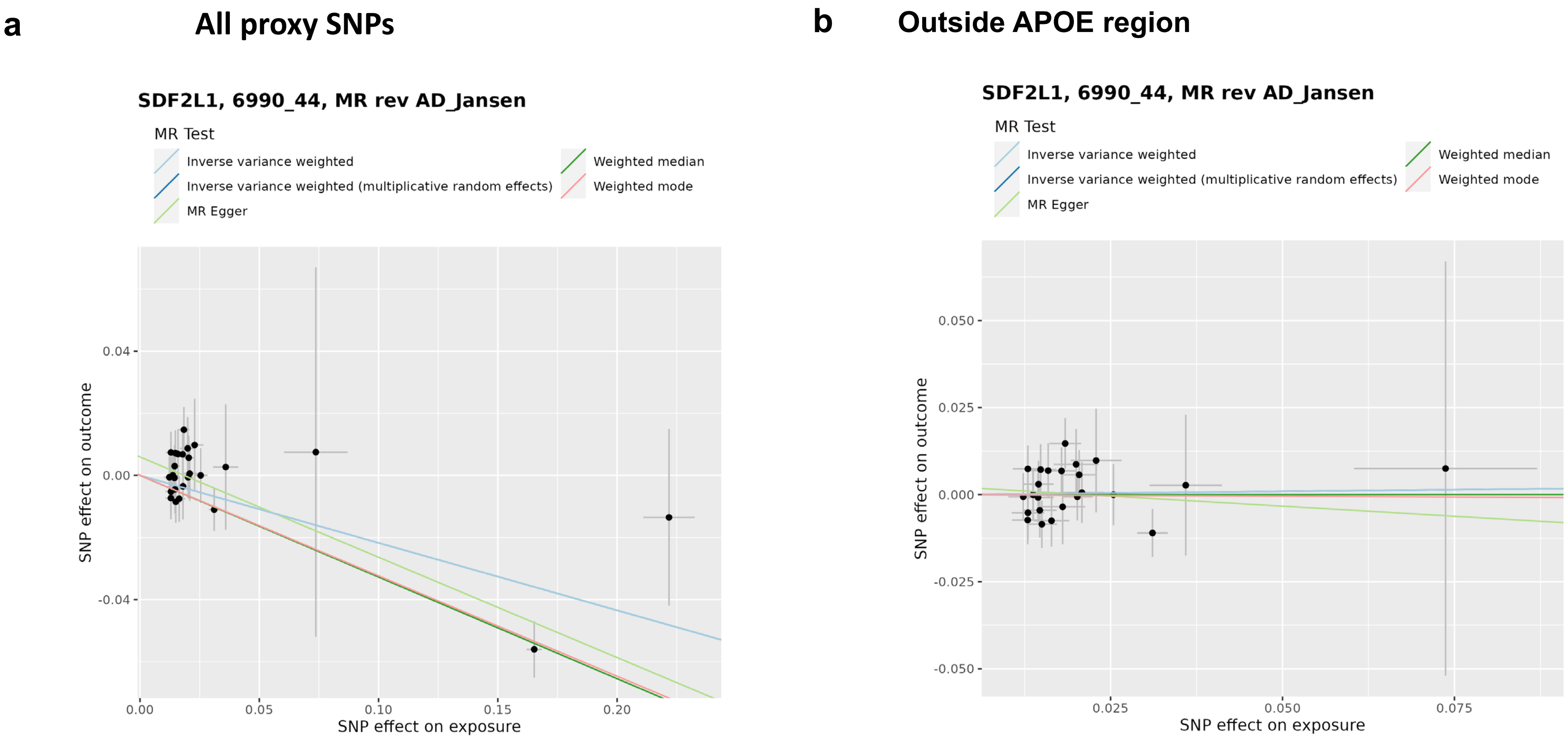

Supplementary figure 31. Scatter plots of proxy SNP effect sizes in the two-sample Mendelian randomization analysis using Alzheimer's disease (AD) susceptibility (Jensen et al. 2019 stage 3 dataset) as the exposure and circulating SDF2L1 as the outcome. Panel a included all proxy SNPs. Panel b included only those outside of the APOE region. The APOE region is defined as 250 Kb on both sides of the APOE gene. The error bars represent the 95% confidence intervals of the SNP effect sizes on the exposure or the outcome.
