# Supplementary material for: Identification of circulating proteins associated with general cognitive function among middle-aged and older adults
Source: Commun Biol. 2023 Nov 3;6:1117. doi: 10.1038/s42003-023-05454-1 (PMC10624811; doi:10.1038/s42003-023-05454-1)
Supplement: Supplementary file 3 — Description of Additional Supplementary Files [file 42003_2023_5454_MOESM3_ESM.pdf]

Description of Additional Supplementary Files

File name: Supplementary Data 1
Description: Characteristics of the discovery cohorts.

File name: Supplementary Data 2
Description: Protein assay platforms among discovery and replication cohorts.

File name: Supplementary Data 3
Description: Characteristics of replication cohorts using the SomaScan platform.

File name: Supplementary Data 4
Description: Characteristics of replication cohorts using the Olink platform.

File name: Supplementary Data 5
Description: Pearson correlations between cognitive scores used in generating general cognitive function in the discovery cohorts.

File name: Supplementary Data 6
Description: Variance explained by the first unrotated principal component (PC1) for the input cognitive scores.

File name: Supplementary Data 7
Description: Summary statistics of general cognitive function represented by the first unrotated principal component (PC1) of cogntive scores from ≥ 3 different domains.

File name: Supplementary Data 8
Description: Bonferroni-corrected significance thresholds in analyses.

File name: Supplementary Data 9
Description: Circulating proteins that were significant in the discovery meta-analyses.

File name: Supplementary Data 10
Description: DSST summary statistics from discovery and replication cohorts.

File name: Supplementary Data 11
Description: Correlations between betas from each discovery cohort for the proteins that were significant in the discovery analysis.

File name: Supplementary Data 12
Description: Protein measures that were significantly associated with general cognitive function in the discovery study and were also associated with related outcomes.

File name: Supplementary Data 13
Description: SNPs that had p<5e-8 in the GWAS of general cognitive function in Davies et al. 2018 and were near the promoter (500kb on both sides) on the encoding gene of a protein that were significantly associated with general cognitive function in the discovery study.

File name: Supplementary Data 14
Description: Among the 246 protein measures that were significant in at least one of the discovery meta-analysis, Spearman correlations between measures from SomaScan and Olink for 99 proteins were available from Pretzner et al. Nat Comms 2019.

File name: Supplementary Data 15
Description: Proteins replicated at FDR < 0.05 for general cognitive function among aged ≥ 25 in SomaScan replication analysis.

File name: Supplementary Data 16
Description: Proteins replicated at FDR < 0.05 for general cognitive function among aged ≥ 65 in SomaScan replication analysis.

File name: Supplementary Data 17
Description: Proteins replicated at FDR < 0.05 for DSST among aged ≥ 65 in SomaScan replication analysis.

File name: Supplementary Data 18
Description: Olink replication results.

File name: Supplementary Data 19
Description: Association between circulating proteins and general cognitive function among participants aged ≥ 65 with the addition of eGFR as covariates.

File name: Supplementary Data 20
Description: Association between circulating proteins and DSST among participants aged ≥ 65 with the addition of eGFR as covariates.

File name: Supplementary Data 21
Description: GO gene sets over-represented for cognition-associated proteins based on the discovery meta-analysis of general cognitive function among participants aged ≥ 25.

File name: Supplementary Data 22
Description: GO gene sets over-represented for cognition-associated proteins based on the discovery meta-analysis of general cognitive function among participants aged ≥ 65.

File name: Supplementary Data 23
Description: GO Gene sets enriched in the two tails of the effect size distribution of the discovery meta-analysis results of general cognitive function among participants aged ≥ 65.

File name: Supplementary Data 24
Description: GO Gene sets enriched in the two tails of the effect size distribution of the discovery meta-analysis results of DSST among participants aged ≥ 65.

File name: Supplementary Data 25
Description: Proteins with significant effect on general cognitive function based on the primary methods of Mendelian randomization (MR) analysis using GWAS summary statistics from Davies et al. 2018.

File name: Supplementary Data 26
Description: Heterogeneity of protein proxy SNPs used in forward MR in the meta-analysis of pQTLs.

File name: Supplementary Data 27
Description: Lookup of significant associations between the proxy SNPs of proteins implicated as affecting general cognitive function with the expression of nearby genes in GTEx 8.0.

File name: Supplementary Data 28
Description: Posterior Probabilities from colocalization analysis of protein and outcome significant in forward MR analysis on AD and general cognitive function.

File name: Supplementary Data 29
Description: Proteins implicated as consequences of general cognitive function: results from Mendelian randomization (MR) analysis using GWAS summary statistics from Davies et al. 2018.

File name: Supplementary Data 30
Description: Heterogeneity of proxy SNPs of general cognitive function or AD used in reverse MR in the meta-analysis of the pQTLs.

File name: Supplementary Data 31
Description: The protein with significant effect on AD susceptibility from Mendelian randomization (MR) analysis using three AD GWAS datasets. The proxy SNP was rs440277 and the MR method was Wald ratio.

File name: Supplementary Data 32
Description: Results of the lookup for significant association of gene expression of cis-genes of the proxy SNP of NECTIN2 (rs440277) in GTEx version 8.0.

File name: Supplementary Data 33
Description: Effect of SVEP1 on AD susceptibility from Mendelian randomization analysis.

File name: Supplementary Data 34
Description: Lead SNP pairs from the colocalization analysis using SuSIE for NECTIN2 and Alzheimer disease (AD).

File name: Supplementary Data 35
Description: Proteins implicated as consequences of AD susceptibility: results from primary methods of Mendelian randomization (MR) analysis using GWAS summary statistics of Kunkle et al. 2019 along with assessment of heterogeneity and pleiotropy based on Egger intercept.

File name: Supplementary Data 36
Description: Proteins implicated as consequences of AD: results from primary and secondary methods using GWAS summary statistics of AD (Kunkle et al. 2019).

File name: Supplementary Data 37
Description: Correlation between proteins that were significantly implicated as affected by AD susceptibility.

File name: Supplementary Data 38
Description: Proteins implicated as consequences of AD susceptibility: results from primary methods using GWAS summary statistics of Jansen et al. 2019 along with assessment of heterogeneity and pleiotropy based on Egger intercept.

File name: Supplementary Data 39
Description: Proteins implicated as consequences of AD susceptibility: results from primary and secondary methods using GWAS summary statistics of Jansen et al. 2019.

File name: Supplementary Data 40
Description: Association between APOE4 carrier status and proteins implicated as consequence of AD susceptibility.

File name: Supplementary Data 41
Description: Association between APOE Ɛ4 risk variants and C-reactive protein in GWAS studies that included UK Bank. These results were obtained from the GWAS catalog.
