# Supplementary material for: Identification of circulating proteins associated with general cognitive function among middle-aged and older adults
Source: Commun Biol. 2023 Nov 3;6:1117. doi: 10.1038/s42003-023-05454-1 (PMC10624811; doi:10.1038/s42003-023-05454-1)
Supplement: Supplementary file 5 — Reporting Summary [file 42003_2023_5454_MOESM5_ESM.pdf]

nature portfolio

Corresponding author(s): Adrienne Tin

Last updated by author(s): Apr 26, 2023

Reporting Summary

Nature Portfolio wishes to improve the reproducibility of the work that we publish. This form provides structure for consistency and transparency in reporting. For further information on Nature Portfolio policies, see our Editorial Policies and the Editorial Policy Checklist.

Statistics

For all statistical analyses, confirm that the following items are present in the figure legend, table legend, main text, or Methods section.

n/a	Confirmed	
☐	☒	The exact sample size (*n*) for each experimental group/condition, given as a discrete number and unit of measurement
☐	☒	A statement on whether measurements were taken from distinct samples or whether the same sample was measured repeatedly
☐	☒	The statistical test(s) used AND whether they are one- or two-sided<br>*Only common tests should be described solely by name; describe more complex techniques in the Methods section.*
☐	☒	A description of all covariates tested
☐	☒	A description of any assumptions or corrections, such as tests of normality and adjustment for multiple comparisons
☐	☒	A full description of the statistical parameters including central tendency (e.g. means) or other basic estimates (e.g. regression coefficient) AND variation (e.g. standard deviation) or associated estimates of uncertainty (e.g. confidence intervals)
☒	☐	For null hypothesis testing, the test statistic (e.g. *F*, *t*, *r*) with confidence intervals, effect sizes, degrees of freedom and *P* value noted<br>*Give P values as exact values whenever suitable.*
☒	☐	For Bayesian analysis, information on the choice of priors and Markov chain Monte Carlo settings
☒	☐	For hierarchical and complex designs, identification of the appropriate level for tests and full reporting of outcomes
☐	☒	Estimates of effect sizes (e.g. Cohen's *d*, Pearson's *r*), indicating how they were calculated

Our web collection on statistics for biologists contains articles on many of the points above.

Software and code

Policy information about availability of computer code

Data collection	A analysis plan with example code and data were distributed to each discovery and replication cohort for conducting the analysis on the association between each protein and cognitive function. These materials can be downloaded from https://drive.google.com/drive/folders/1EIj7E5-e032AhjP8lYth7Q6qKvLdklDc?usp=share_link
Data analysis	The R packages used were metafor version 3.0.2 for the meta-analysis of the association between each protein and cognitive function, TwoSampleMR version 0.5.4 for the Mendelian randomization analysis, coloc version 5.1.0.1 for colocalization analysis, and clusterProfiler version 3.16.1 for enrichment analysis.

For manuscripts utilizing custom algorithms or software that are central to the research but not yet described in published literature, software must be made available to editors and reviewers. We strongly encourage code deposition in a community repository (e.g. GitHub). See the Nature Portfolio guidelines for submitting code & software for further information.

Data

Policy information about availability of data

All manuscripts must include a data availability statement. This statement should provide the following information, where applicable:

- Accession codes, unique identifiers, or web links for publicly available datasets
- A description of any restrictions on data availability
- For clinical datasets or third party data, please ensure that the statement adheres to our policy

The summary statistics of the discovery and replication meta-analysis of the association between circulating protein and cognitive function will be available in figshare. The datasets used in Mendelian randomization were download from the studies that published those results: deCODE protein quantitative trait loci (pQTL) dataset https://www.decode.com/summarydata/, Interval pQTL dataset: the European Genotype Archive (accession number EGAS00001002555), the Fenland pQTL dataset: https://gwas.mrcieu.ac.uk/ and https://www.ebi.ac.uk/gwas/, the GWAS of Alzheimer's disease by Kunkle et al.: The National Institute on Aging Genetics of Alzheimer's Disease Data Storage Site (NIAGADS) accession NG00075, the GWAS of Alzheimer's disease by Jansen et al.: https://ctg.cncr.nl/software/summary_statistics. The gene expression quantitative trait loci (eQTL) data were download from the Genotype-Tissue Expression (GTEx) portal https://gtexportal.org/home/datasets, the GWAS of general cognitive function: requests can sent to the chairs of the CHARGE and COGENT consortia.

Research involving human participants, their data, or biological material

Policy information about studies with human participants or human data. See also policy information about sex, gender (identity/presentation), and sexual orientation and race, ethnicity and racism.

Reporting on sex and gender	Biological sex was used as a covariate in the analysis of the association between protein and cognitive function.
Reporting on race, ethnicity, or other socially relevant groupings	Self-reported race was used as a covariate in the analysis of the association between protein and cognitive function.
Population characteristics	Population characteristics of each participating cohort are reported in Supplementary Tables 1, 2, 3 and 4.
Recruitment	All participating cohorts are population-based. The details of recruitment criteria are reported in Supplementary Methods.
Ethics oversight	The protocols of all participating cohorts were approved by their respective Institutional Review Boards.

Note that full information on the approval of the study protocol must also be provided in the manuscript.

Field-specific reporting

Please select the one below that is the best fit for your research. If you are not sure, read the appropriate sections before making your selection.

☒ Life sciences ☐ Behavioural & social sciences ☐ Ecological, evolutionary & environmental sciences

For a reference copy of the document with all sections, see nature.com/documents/nr-reporting-summary-flat.pdf

Life sciences study design

All studies must disclose on these points even when the disclosure is negative.

Sample size	The sample size of each participating cohort are reported in Supplementary Tables 1, 2, 3 and 4.
Data exclusions	Participants excluded were those with prevalent dementia or stroke or without data in cognitive function, protein measures, or covariates.
Replication	The association of the significant proteins in the discovery analysis were evaluated in independent cohorts for replication.
Randomization	We conducted an observational study, which does not involved randomization.
Blinding	We conducted an observational study without any intervention, therefore blinding is not applicable.

Reporting for specific materials, systems and methods

We require information from authors about some types of materials, experimental systems and methods used in many studies. Here, indicate whether each material, system or method listed is relevant to your study. If you are not sure if a list item applies to your research, read the appropriate section before selecting a response.

Materials & experimental systems

n/a	Involved in the study	
☒	☐	Antibodies
☒	☐	Eukaryotic cell lines
☒	☐	Palaeontology and archaeology
☒	☐	Animals and other organisms
☒	☐	Clinical data
☒	☐	Dual use research of concern
☒	☐	Plants

Methods

n/a	Involved in the study	
☒	☐	ChIP-seq
☒	☐	Flow cytometry
☒	☐	MRI-based neuroimaging
